# Land‐use change promotes avian diversity at the expense of species with unique traits

**DOI:** 10.1002/ece3.2389

**Published:** 2016-10-05

**Authors:** Bernard W. T. Coetzee, Steven L. Chown

**Affiliations:** ^1^ Centre for Invasion Biology Department of Botany and Zoology Stellenbosch University Private Bag X1 Matieland 7602 South Africa; ^2^ School of Biological Sciences Monash University Melbourne Victoria 3800 Australia

**Keywords:** Avifauna, body size, fourth‐corner statistics, land‐use change, multidimensional functional indices, urbanization

## Abstract

Land‐use change may alter both species diversity and species functional diversity patterns. To test the idea that species diversity and functional diversity changes respond in differing ways to land‐use changes, we characterize the form of the change in bird assemblages and species functional traits along an intensifying gradient of land use in the savanna biome in a historically homogeneous vegetation type in Phalaborwa, South Africa. A section of this vegetation type has been untransformed, and the remainder is now mainly characterized by urban and subsistence agricultural areas. Using morphometric, foraging and breeding functional traits of birds, we estimate functional diversity changes. Bird species richness and abundance are generally higher in urban and subsistence agricultural land uses, as well as in the habitat matrix connecting these regions, than in the untransformed area, a pattern mainly driven through species replacement. Functionally unique species, particularly ground nesters of large body size, were, however, less abundant in more utilized land uses. For a previously homogenous vegetation type, declines in the seasonality of energy availability under land‐use change have led to an increase in local avian diversity, promoting the turnover of species, but reduced the abundance of functionally unique species. Although there is no simple relationship between land‐use and diversity change, land‐use change may suit some species, but such change may also involve functional homogenization.

## Introduction

Human population expansion is unlikely to stabilize in the 21st century (Gerland et al. 2014). As the expansion of the human population is associated with land‐use change, the earth's biomes have been so fundamentally altered that they are now best characterized as “anthropogenic biomes” (Ellis and Ramankutty 2008). Much attention has therefore been focused on the impacts of human land‐use change on biodiversity (Gaston 2000; Gaston et al. 2003; Dobrovolski et al. 2013), given that land‐use change can promote biodiversity declines (Foley et al. 2007). Understanding the impact of urban and agricultural intensification on biodiversity is of particular importance, as they form a substantial component of human land‐use activities. From 1700 to 2000, agriculture and urban settlement increased from 5% to 39% of the earth's total ice‐free terrestrial surface (Ellis et al. 2010). Both urbanization (Evans et al. 2011) and agriculture (Tilman 1999) continue to expand rapidly, with a concomitant expected decrease in biodiversity (Vitousek et al. 1997; Foley et al. 2007; Dobrovolski et al. 2013).

Although the impacts of habitat alteration on biodiversity are often revealed through changes in abundance, richness, and assemblage composition (e.g., Van Rensburg et al. 2009; Pautasso et al. 2011; Maron et al. 2013; Rogers and Chown 2014), land‐use change also affects species‐specific functional traits (Peres 2000; Pocock 2011; Luck et al. 2012; Newbold et al. 2013). However, land‐use change might not alter species diversity and functional trait diversity in a similar manner. For example, the species and functional diversity relationship varies with disturbance intensity in Boreal forests trees (Biswas and Mallik 2011) and Australian birds may display striking differences in their response to land use when comparing species or functional diversity across 24 land uses along an intensification gradient (Luck et al. 2013). Indeed, while it is often assumed that functional diversity declines with species diversity decline under land‐use change, there is growing empirical support that changes in species and functional trait diversity may follow various trajectories, as land‐use change impacts primarily on community assembly processes (Mayfield et al. 2010; Cadotte et al. 2011). The variation of such responses to environmental change among species may determine the resilience of communities and ecosystems to environmental degradation, like that of land‐use change (Mori et al. 2013). Thus, an improved understanding of how land‐use change alters species richness and abundances patterns, combined with how it alters the functional diversity of those species assemblages, is essential for understanding and mitigating the impacts of land‐use change on biodiversity (Mayfield et al. 2010; Cadotte et al. 2011; Cardinale et al. 2012; Mori et al. 2013). In consequence, functional trait diversity analyses may be more suitable to assess the impacts of disturbance to ecosystems, as the impacts may be independent of changes in species richness (Mouillot et al. 2013).

Birds make a useful group to investigate the impact of environmental changes such as land‐use change (e.g., Schulze et al. 2004). Indeed, previous work has demonstrated that in urban areas, bird assemblages can show marked changes, typically being dominated by a few highly abundant species that are well adapted to human‐affected landscapes, be they native or alien invasive species (Clergeau et al. 1998; McKinney 2006; Van Rensburg et al. 2009; Evans et al. 2011; de Lima et al. 2013). The functional traits of birds are also affected because foraging guilds, such as granivores, frugivores, and mixed feeders, may benefit in response to favorable anthropogenic alterations of their habitat through urban and agricultural conversion (Clergeau et al. 1998; Child et al. 2009; Greve et al. 2011; Rogers and Chown 2014). Large‐bodied birds, typically raptors, often decline markedly in abundance with landscape transformation, especially outside of protected areas (Herremans and Herremans‐Tonnoeyr 2000; Peres 2000; Devictor et al. 2007; Thiollay 2007).

Over the next three decades, Africa's population expansion is projected to accelerate the most rapidly of all the continents (Gerland et al. 2014). Much of the concomitant land‐use change associated with such expansion is expected in “village biomes,” which are agricultural regions interspersed with urban settlements (Ellis and Ramankutty 2008). The African savanna biome covers much of the continent and also supports a large proportion of its biodiversity, but is changing rapidly given growing human populations and development (Scholtz and Chown 1993; Chown 2010; Trimble and van Aarde 2012). Compared to other biomes, the impacts of land‐use change associated with agricultural intensification and urbanization in African savanna ecosystems are also poorly studied (Trimble and van Aarde 2012).

Here, we therefore investigate how avian assemblage diversity and species functional trait diversity vary across a gradient of intensifying land‐use change in a savanna ecosystem, especially to examine the idea that species and functional diversity responses to land‐use change are variable. We focus our study on an exemplar African savanna ecosystem in and around the Kruger National Park (KNP), South Africa. We sampled within a historically homogeneous vegetation type, a portion of which has been conserved within the KNP and the rest of which has been subjected to a range of land uses. In consequence, changes in bird assemblages should largely reflect anthropogenic alteration to the landscape, rather than confounding variables that could potentially also drive species assemblages change (e.g., Pautasso et al. 2011).

We examine several key ideas. Primarily, we set out to examine the idea that species diversity and functional diversity change in different ways with land‐use change, and that they show complex relationships related (see Mayfield et al. 2010 for rationale). We explore assemblage level changes between land‐use types by comparing community changes using standard metrics, such raw counts, analysis of similarity, nonmetric multidimensional scaling, beta‐diversity change, and by identifying indicator species. Then, using morphometric, foraging and breeding functional traits of birds, we estimate the functional diversity changes between land‐use types. To do so, we use functional diversity indices. Next we use fourth‐corner statistics to test for associations between the abundance structure of the community, functional characteristics of the species (species traits), and environmental conditions (site traits; Dray and Legendre 2008; Neuschulz et al. 2013). This approach helps disentangle the complex relationships among species and functional measures of diversity. To provide further insight into the potential factors influencing these relationships, we also determined the extent to which environmental energy availability may be a potential driver that underlies variation in both species diversity and functional diversity. In particular, we examine the idea that it is not only energy availability, but also its seasonality that influences variation in diversity (see Rohde 1992 for an early discussion of the hypothesis). Environmental energy availability is a significant determinant of especially larger scale variation in species richness, and typically, species richness increases with energy availability (Evans et al. 2005; Storch et al. 2005), and species abundance may also do so (Hurlbert 2004). Land‐use change may directly alter energy availability in terms of primary productivity (Haberl et al. 2007). Despite these relationships, examinations of the importance of seasonality in energy availability are not common, nor are demonstrations that energy availability can also influence functional diversity independently of species diversity variation.

Because impacts of land use on biodiversity may vary regionally, the expected responses of avian assemblages under modified land use are not straightforward to predict, but we expect alterations to species richness, abundance, and functional traits under high land use, in keeping with general global trends (Sinclair et al. 2002; Devictor et al. 2007; Thiollay 2007; Greve et al. 2011). In particular, species with large body size may decline in abundance with landscape transformation (Herremans and Herremans‐Tonnoeyr 2000; Peres 2000; Devictor et al. 2007; Thiollay 2007).

## Methods

### Study region

**Figure 1:**
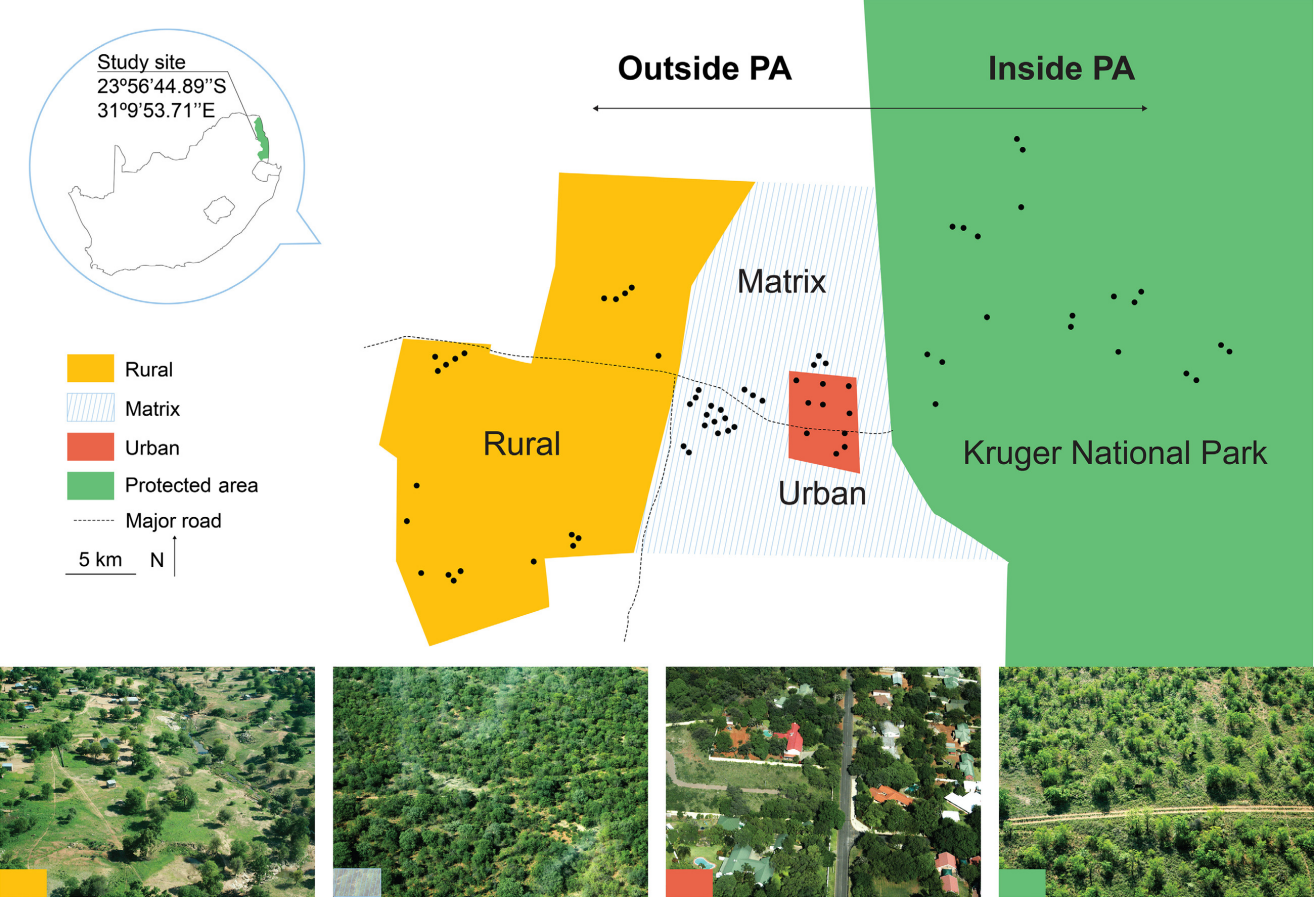
Map of the study region indicating major land‐use types, with aerial photographs of each. Point sampling locations are indicated by filled black circles.

All sampling was conducted in and around the town of Phalaborwa, South Africa (23°56′44.89″S 31°9′53.71″E). Phalaborwa was established in the early 1960s to a serve the nearby copper mining operations, has a population of approximately 140 000, and is characterized by semi‐urban households. All sampling sites were in a historically identical vegetation type, the Phalaborwa Sandy Mopaneveld sensu Mucina and Rutherford (2006). Since the establishment of the town, land‐use change has altered the landscape, and now four land uses characterize this vegetation type: (1) A “protected area,” inside the Kruger National Park (KNP), (2) a “matrix area” representing the habitat matrix between (3) a “rural area” outside KNP, and (4) an “urban area” outside KNP. These classes represent a gradient of land‐use intensity from low (protected‐matrix areas) to high (rural–urban areas; Fig. 1).

The KNP was established in 1931, and although managed, the protected area represents a comparatively pristine ecosystem (du Toit et al. 2003). The rural area is primarily a subsistence‐farming region, where humans living rurally produce crops or raise livestock primarily for their own consumption. This area is under heavy grazing from cattle and goats. The urban area is semi‐urbanized (sensu Pautasso et al. 2011) mainly characterized by suburban houses with large gardens, but also incorporating more transformed sections such as tarred roads and commercial buildings. The matrix area is characterized by currently unused, unfarmed and unprotected land under low extractive use with minimal grazing impact and is not under any formal designation of traditional land‐use classes (see illustrative aerial photographs in Fig. 1).

### Study design

We followed an exact distance point transect sampling approach (Thomas et al. 2010). Point counts were located at random in each land use: protected area (*n* = 20), rural area (*n* = 20), matrix area (*n* = 20), and urban area (*n* = 10; due to its small spatial extent). Extensive obstructions (roads, fences, private lands, mining operations, settlements, and safety concerns) prevented true random placement of points outside the protected area, but in those cases, they were relocated to the nearest point that met the sampling criteria. Points in the rural area were located in grazing lands away from dense human settlements. Points were at least 400 m from conspicuous boundaries (major roads, boundaries between land use, any water body), and at least 300 m apart to avoid pseudoreplication (Sutherland 1996; Van Rensburg et al. 2009; Greve et al. 2011). Typically, points were much further apart ($\overset{¯}{x}$ = 11.95 km; SD = 7.7 km; *n* = 70).

Birds were counted (by one observer [BWTC]) at point stations for 10 min, which commenced after a two‐minute period to acclimatize birds. All birds seen and heard were noted, and the distance from observer recorded with a laser rangefinder, up to 100 m. Flying birds were omitted. Surveys were conducted between 06:00 and 10:00 during peak bird activity and only in good weather (no heavy wind or rain).

Sampling was conducted over 2 years (2010, 2011) in February–March and October–November (thus four sampling events in total), when most migrant species actively utilize landscapes in the region (Hockey et al. 2005). Points were visited twice per sampling event (thus 560‐point counts conducted), but data for such repeat counts per sampling event were pooled, resulting in a total of 280‐point counts over all four sampling events (70‐point counts with pooled data for each of the four sampling events).

### Data analysis: data preparation

All analyses are reported with point count data combined for each land use across years and months, because a generalized linear model [assuming a Poisson distribution, corrected for overdispersion, with a log link function fitted in R (R Core Team 2014)] revealed that only land use, and not sampling year or month, had a significant effect on species richness or abundance (Table S1).

The sampling adequacy of our study design to capture the potential species present was assessed with EstimateS v. 8.20, before data analysis (Colwell 2005). Because sample‐based rarefaction approached an asymptote (Fig. S1), singleton species additions may be expected with further sampling, but observed species richness can nonetheless be considered a robust and comparable approximation across land uses (Colwell 2005).

Because differences in species detectability may bias density estimates, we tested the influence of differences in species detectability before data analysis (Thomas et al. 2010; Greve et al. 2011). Due to low sample sizes of rarer species, as is typical in community ecology, detection functions could not be fitted to all species, or fitted to species from individual land uses, and so we opted for a surrogate species approach, following others (Thomas et al. 2010; Greve et al. 2011; and see Mulwa et al. 2012). Surrogate species groups were identified with a trait dendrogram based on a hierarchical cluster analysis on a dissimilarity matrix (details follow under “Data analysis: trait changes”). When correcting for detection across the seven emergent species functional groups (see Fig. S2; Table S2), a high correlation remained between observed density, and density corrected for detection (Fig. S3; Pearson's *r* = 0.994; *P* < 0.001). For each group, the best‐fit detection function was also a monotonically decreasing detection function at a 40‐m truncation (Fig. S4). Consequently, we consider the influence of species detectability and detectability between land uses negligible in our study, and so densities were not adjusted for detectability (following Thomas et al. 2010; Greve et al. 2011). As such, all further analyses are conducted on species included at a 40‐m truncation.

### Data analysis: assemblage changes

To investigate whether significant differences exist in bird assemblages across and between land use, we compared bird assemblages using an analysis of similarity (ANOSIM) and nonmetric multidimensional scaling plots (nMDS; PRIMER v.5; Clarke and Warwick 2001). An ANOSIM broadly analyses the difference in overall assemblage structure, where the closer a significant Global *R* statistic is to one, the more distinct the differences. An nMDS displays the relationships between assemblages based on the similarity matrix; the closer the data points are the more similar they are (Clarke and Warwick 2001). Rare and common species were weighted equally by square root transformation of the data before analysis, and a Bray–Curtis similarity measure was used to calculate the similarity matrix (Clarke and Warwick 2001). The characteristic and common species from all land use were identified using the indicator value method (IndVals; Dufrene and Legendre 1997). Raw abundances for the most prevalent and characteristic species were plotted with the “mvabund” package (Wang et al. 2012) in R (R Core Team 2014). The extent of spatial turnover of species along the land‐use gradient was quantified with beta‐diversity metrics. Beta‐diversity patterns can be partitioned into dissimilarity in communities due to species replacement (Simpson dissimilarity or spatial turnover, ß_sim_) and dissimilarity due to nestedness (nestedness‐resultant dissimilarity, ß_sne_, Baselga 2012). We calculated beta diversity in the “betapart” package (Baselga and Orme 2012) and illustrated these diversity metrics using a hierarchical cluster dendrogram in R (R Core Team 2014).

### Data analysis: trait changes

To identify those traits which most strongly respond to changing land use, species functional traits were captured in three classes: morphometric, foraging guilds, and nesting traits. Morphometric traits were chosen that are inferred to have strong relationships with avian life history. These were mean: body mass, tarsus, wing, culmen, and tail length from Hockey et al. (2005). Foraging guilds designation followed Greve et al. (2011): frugivores, granivores, insectivores, nectarivores, mixed (more than one foraging guild), and predators (of vertebrates). Nesting traits were the mean clutch size and egg length, and nest type as a categorical variable (either hole, cup, oval, platform, or ground nester, and brood parasite), from Hockey et al. (2005).

We tested for potential change of traits across the landscape in two ways: by identifying emergent functional groups and by relating species traits to land uses. First, to identify the major functional groups and their abundance change in our study region from the entire species assemblages (also used to test for differences in detectability, above), we constructed a trait dendrogram using the “GFD” function in R (Mouchet et al. 2008; R Core Team 2014). Functional groups are identified with a trait dendrogram based on a hierarchical cluster analysis from a dissimilarity matrix, built with the species traits described (Mouchet et al. 2008). Because clustering approaches differ in their ability to represent the distribution of species in a functional space, we used a consensus tree across clustering algorithms (see Mouchet et al. 2008). By combining the species abundance data with the seven emergent groups, we calculated the mean and standard deviation of abundance for each functional group per land use. We also examined the changes in the body size of species assemblages between land uses with body size frequency distribution (BSFD) histograms using body mass in grams for all species (Coetzee et al. 2013).

To characterize functional diversity (FD) for assemblages themselves across different land uses (the diversity of species traits in ecosystems; Schleuter et al. 2010), we used multidimensional functional indices. Villéger et al. (2008) introduced FD indices that disaggregate FD into components of functional richness (FRic; the amount or “volume” of functional space filled by an assemblage), functional evenness (FEve; the evenness of abundance distribution in a functional trait space), and functional divergence (FDiv; the spread of abundance along a functional trait axis). These indices are independent of species richness, and so represent indices of the trait variation between land uses (Villéger et al. 2008). To downweigh the influence of highly abundant species, data to calculate functional diversity were the species by traits matrix and species presence absence data, per land use and was performed in the “FD” package (Laliberté and Shipley 2013) in R (R Core Team 2014).

Second, we related species traits to different land uses using fourth‐corner statistics. The approach can identify positive or negative associations between the biological or other traits of organisms and the environmental characteristics of the locations at which they are found (Dray and Legendre 2008), as well as the statistical strength of that association (Brown et al. 2014). In consequence, the method can uncover how land‐use change selects for particular species traits at that location, and so results of the fourth‐corner analysis can identify how the abundances of different traits vary as land‐use varies (Dray and Legendre 2008; Brown et al. 2014). The method links three data matrix tables: a table “L” with presence values for species across points, a table “R” with variables describing the traits of land cover areas (environmental conditions), and a table “Q” containing traits (e.g., morphological, breeding, and foraging attributes) of the species (Dray and Legendre 2008). To implement this test examining which species traits are associated with land uses, we used the “trait.mod” function in R (Brown et al. 2014; R Core Team 2014). Data for environmental traits were the presence or absence of land‐use categories (protected, matrix, rural, or urban areas).

### Data analysis: energy availability

To test the influence of energy availability in structuring communities (here measured as primary productivity), we used the Normalised Difference Vegetation Index (NDVI; NASA 2014). NDVI is calculated from the red and near‐infrared reflectance of vegetation, and so provides a proxy of primary productivity and vegetation structure, both potential drivers of bird diversity (Kerr and Ostrovsky 2003; Rogers and Chown 2014; Nieto et al. 2015). Mean NDVI was calculated at a 250‐m^2^ resolution over a 16‐day period for both the February and November 2010–2011 sampling periods and averaged between February and November. NDVI data were then extracted for each sampling point location using ArcGIS 10.2 (ESRI 2014). Because seasonality in energy availability, or temporal heterogeneity, is being increasingly recognized as a factor influencing diversity (Menge and Sutherland 1976; Rohde 1992; Archibald et al. 2010; Stein et al. 2014), we also considered it explicitly as follows. In our study region, the peak‐growing season is in February. The lowest point in the growing season is in late winter (August), before the onset of the wet season typically starting September–October (see Fig. S5). To investigate seasonal differences in NDVI across land use, we therefore calculated the difference between February and August NDVI for each point location, determined as for primary productivity above.

To assess the influences of energy availability for bird species richness and abundance, we used generalized linear models (GLMs), assuming a Poisson distribution, corrected for overdispersion, with a log link function, fitted in R (R Core Team 2014). Explanatory variables were mean NDVI, the seasonal change in NDVI (the difference between February and August NDVI). In order to model the potential spatial dependence between observations, we included latitude and longitude as response variables. Functional diversity may also vary with energy availability if an increase in productivity in a landscape promotes a greater diversity of functional groups. To assess the influences of energy availability on the response variables of bird functional richness, diversity, and evenness, we fit identical GLMs as described above.

## Results

### Assemblage changes

**Figure 2:**
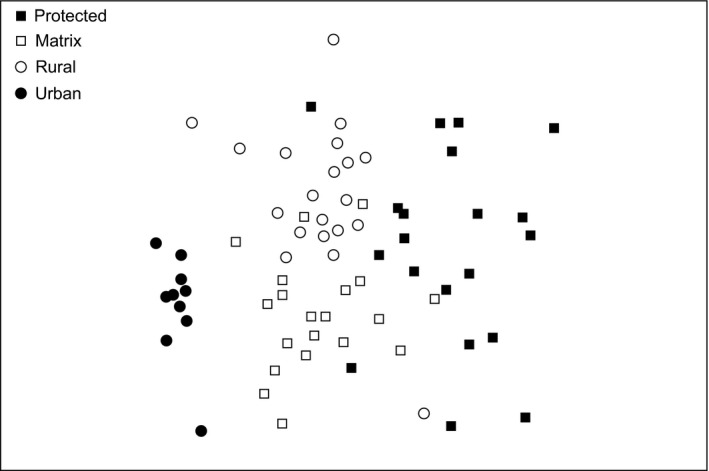
Nonmetric multidimensional scaling plot of bird assemblages inside Kruger National Park (filled squares), and outside the protected area in rural (open circles) and urban (filled circles) land uses, and the connecting matrix (open squares). The greater the distance between sampling points, the greater the differences in their assemblages are. In terms of their overall assemblage structure, apart from urban, land uses are overlapping and only somewhat different (ANOSIM; Global *R *=* *0.44, *P *=* *0.01; Stress = 0.26).

A total of 2382 individuals, representing 106 species, were recorded. Species richness and abundance were highest in the matrix, rural, and urban areas (Table 1). A unique assemblage, indicated by clustering in a multidimensional scaling plot, characterized the urban area, but assemblage differences were not as pronounced across the other land uses (Fig. 2; ANOSIM = Global *R *=* *0.44, *P *=* *0.01). The protected area contained fewer individuals, but greater abundances of some species than the other areas, such as the Red‐billed Quelea (*Quelea quelea*; see Appendix S1; all species detailed in Hockey et al. 2005). All land uses contained the ubiquitous Laughing Dove (*Streptopelia senegalensis*), but the urban area contained six times as many individuals of this species on average than other land uses (Appendix S1; Fig. S6). When considering raw abundances, mixed feeders such as the Red‐eyed Dove (*Streptopelia semitorquata*), Yellow‐Fronted Canary (*Crithagra mozambicus*), Bronze Mannikin (*Spermestes cucullatus*), and Southern Masked Weaver (*Ploceus velatus*) were most abundant in the urban area (Fig. S6). Insectivore abundance within the urban area was particularly high, especially for indicator species such as the African Paradise Flycatcher (*Terpsiphone viridis*) and Kurrichane Thrush (*Turdus libonyanus*; Table S3; Appendix S1); however, the Rattling Cisticola (*Cisticola chiniana*), an indicator species elsewhere, was absent from all urban points (Table S3; Appendix S1).

**Table 1:** Total species richness and abundance, and mean species richness and abundance, respectively, together with two richness estimators: Jacknife2 (obtained without re‐sampling) and Chao1, across sampling sites in all land uses

	Protected (*n* = 20)	Matrix (*n* = 20)	Rural (*n* = 20)	Urban (*n* = 10)
Total species rich.	48	65	58	59
Total species abund.	387	621	517	857
Mean species rich. (SD)	7.7 (2.72)	13.65 (3.5)	10.3 (3.18)	21.9 (5.65)
Mean abund. (SD)	19.35 (27.48)	31.05 (13.26)	25.75 (7.86)	85.7 (22.84)
Mean Jacknife2	79.18	94.47	104.98	82.77
Chao1 (95% CI)	61.13 (51.88; 92.38)	80.11 (69.76; 112.96)	72.15 (63.38; 98.48)	70.14 (62.06; 99.63)

Abund, Species abundance; SD, Standard deviation; CI, Confidence intervals.

Because the protected and matrix areas have a lower diversity of species, the beta diversity due to species turnover (ß_sim_) was highest between those regions and the rural area. Species turnover was also highest between protected‐matrix‐rural areas and the urban area (Fig. S7A). Dissimilarity due to nestedness was greatest between matrix and urban areas (Fig. S7B; ß_sne_), indicating that the urban area is not merely a nested subset of the matrix area, but contains species not found elsewhere in the study site.

### Trait changes

**Figure 3:**
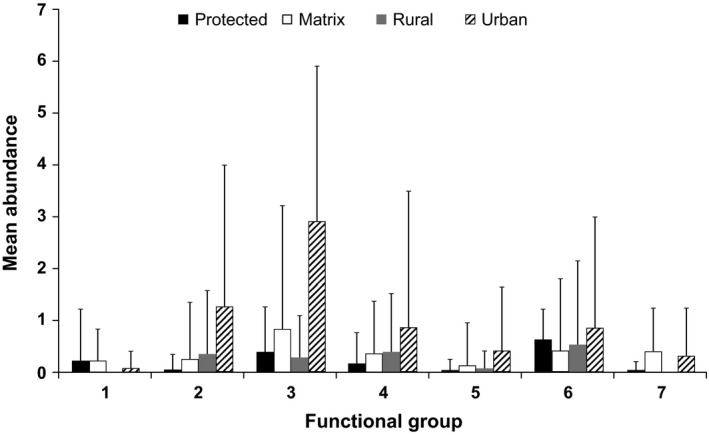
Mean number of species per point count in seven functional groups, across protected, rural, urban, and matrix land‐use areas. Groups were identified using a trait dendrogram on morphometric, breeding, and foraging traits (Fig. S2). Bars indicate standard deviations. Functional groups are 1: large mixed feeders and ground nesters; 2: small mixed feeders, frugivores, and hole nesters; 3: small‐medium granivores and platform nesters; 4: small‐medium insect‐ and nectarivores, cup nesters, and parasitic breeders; 5: small‐medium mixed feeders and breeders; 6: small granivores and cup nesters; 7: large mixed feeders and hole nesters. See Table S2 for exemplar species.

Functionally unique groups identified by the dendrogram, particularly large‐bodied ground nesters (Group 1), showed a decline in more intensely utilized land uses, while smaller bodied mixed feeders, insectivores, and canopy nesters greatly increased (groups 2–5), especially in the urban region (Fig. 3 and see Fig. S2; Table S2). Larger species with specific bill morphology reflecting their foraging requirements (e.g., insectivorous/omnivorous hornbills – Group 7), reached their highest diversity in the matrix area.

Similar to species richness, functional richness increased markedly outside the protected area, as more species from a variety of functional groups are added to the assemblages in the matrix, rural, and urban areas (Tables 1 and 2; Fig. 3), mainly driven by species replacement (see Fig. S7). Functional richness was particularly high in the rural area, which implies that its assemblage had a high diversity of functional traits, despite the absence of species with unique traits found in other land uses, such as large ground nesters (Group 1; Fig. 3) and hornbills (Group 7; Fig. 3). Functional evenness however was highest in the protected and matrix areas, and higher in the urban than in the rural area, indicating that the species in those assemblages occupied similar trait spaces at similar abundances and so are not dominated by a single species. Functional divergence, the spread of abundance along a functional trait axis, was highest in the protected area, driven in part by large‐bodied and morphologically differentiated species (Table 2; e.g., hornbills; large ground nesters, see Figs. 3 and S2). The high functional divergence in the protected area indicated that the most abundant species there have extreme functional traits when compared to the functional trait space occupied by that assemblage. Functional divergence was identical in matrix and urban areas.

**Table 2:** Functional diversity decomposed into constituent parts of functional richness, evenness, and divergence

	Protected	Matrix	Rural	Urban
Functional richness (FRic)	4.11	8.21	12.46	10.00
Functional evenness (FEve)	0.61	0.64	0.49	0.56
Functional divergence (FDiv)	0.89	0.74	0.69	0.74

**Figure 4:**
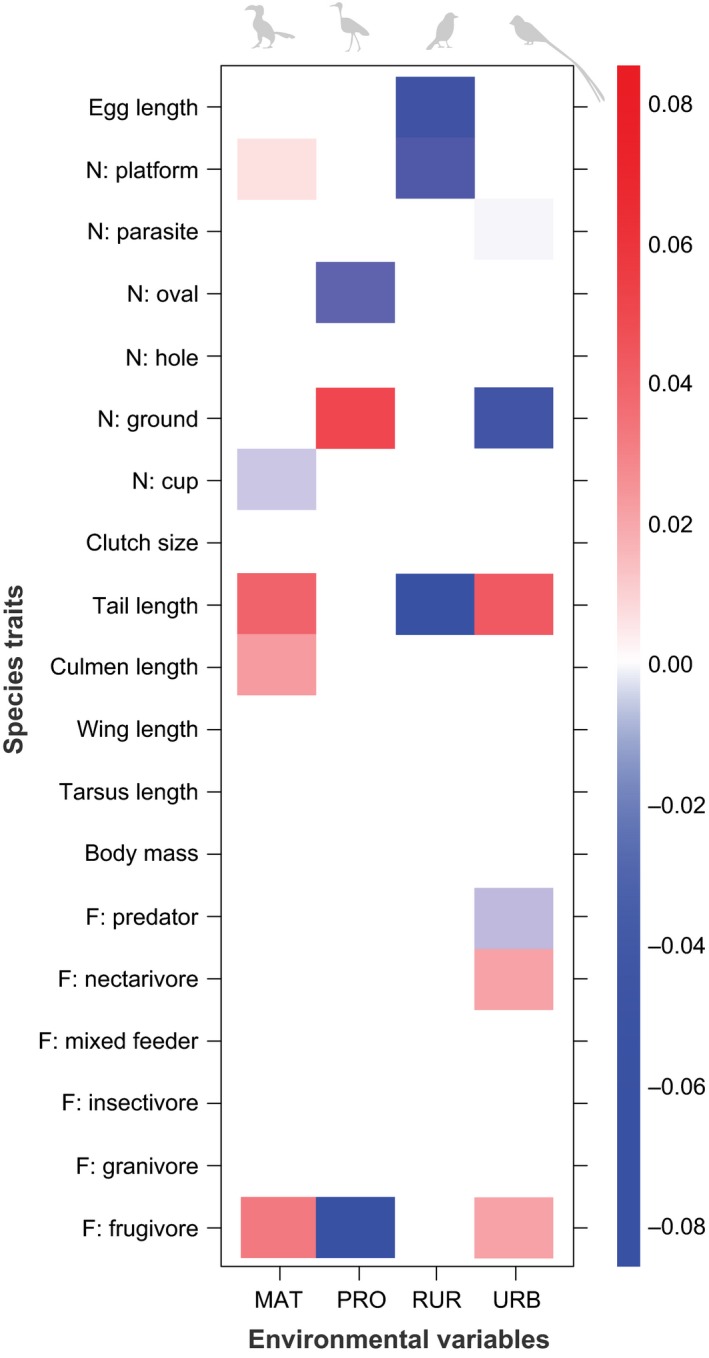
Fourth‐corner statistics results for species presences across species traits and land‐use categories. Brighter squares show stronger associations than paler ones, positive associations are red, and negative associations are blue. Land uses run in columns, while species traits are in rows. See Results for details on interpretation. MAT = matrix; PRO = protected; RUR = rural, URB = urban land‐use areas. *N* = nest, F = foraging guild.

The sign and magnitude of the interaction coefficients denoted the nature and strength of the association of the trait–environment relationship, respectively (Brown et al. 2014). For example, there was a positive and strong association between the presence of ground nesters in the protected area, and conversely, a negative and strong association between the absence of ground nesters in the urban area (Fig. 4). The only morphometric traits that showed an association with land uses were tail, culmen, and egg length. The matrix area has the highest abundance of hornbills (Fig. 3), which had long culmen and tail lengths, and contributes to driving the positive and strong association of those traits and that region (Fig. 4). Assemblages in the rural area were characterized by traits such as smaller eggs and shorter tails (thus species of smaller body size) and nonplatform nesters (Fig. 4). There was a reduced abundance of frugivores in the protected area (see Fig. 4), but frugivores showed a positive and strong association with both matrix and urban land areas, while nectarivores only showed positive association with the urban area. Together with evidence of high abundances (Table 1), but low body mass (Fig. S8) of species outside the protected area, the finding from the fourth‐corner analysis suggested a decline of large‐bodied species outside of the protected area. Indeed, the seven largest species in our study area are ground nesters (such as francolins and guineafowl) and hornbills (their mean mass in grams $\overset{¯}{x}$ = 500; SD = 401.1; *n* = 7, mean mass for all species $\overset{¯}{x}$ = 71.9; SD = 153; *n* = 106), and it is these large‐bodied species that are absent from rural land use (Table S1; Fig. S2).

### Energy availability

Both species richness and abundance had significant positive relationships with mean NDVI across protected, matrix, rural, and urban areas, as well as a significant negative relationship with seasonal NDVI (Table 3). These models explained 46.8% and 35.5% of variance in species richness and abundance, respectively. Although species richness and functional diversity are not correlated in our study (Pearson's *r* = 0.22; *P* = 0.06; *N* = 70), GLMs indicated that functional richness also had significant positive relationship with mean NDVI, as well as a significant negative relationship with seasonal NDVI (Table S4). The model explained 22.8% of variance in functional richness. Neither functional evenness nor divergence displayed a significant relationship with either mean NDVI or seasonal NDVI (Table S4).

**Table 3:** Results from a general linear model comparing both species richness and abundances across all land uses as a function of primary productivity explanatory variables (mean NDVI and seasonal NDVI), latitude, and longitude

Species richness	Deviance explained		46.8%
Variables	Coefficient	Standard error	*P*
Intercept	−51.75	53.68	0.34
Mean NDVI	0.0005	7.57	<0.001*
Seasonal NDVI	−0.0004	5.90	<0.001*
Latitude	−2.11	1.60	0.19
Longitude	0.07	0.68	0.92

Abundance	Deviance explained		35.5%
Variables	Coefficient	Standard error	*P*
Intercept	55.99	115.73	0.63
Mean NDVI	0.0005	0.0001	<0.001*
Seasonal NDVI	−0.0006	0.0001	<0.001*
Latitude	1.05	3.47	0.76
Longitude	−0.92	1.47	0.53

Significant *P* values are denoted with an asterisk.

## Discussion

We aimed to determine the changes in avian assemblage structure and changes in the species traits of those communities, as a consequence of land‐use change outside a protected area. To our surprise, land‐use change outside the protected area generally increased both avian species richness and abundance. The urban area in particular contained a relatively unusual assemblage when compared to the other land uses, as shown by high species turnover between this area and others. It was also characterized by greatly increased abundances of some species common to all land uses, such as the doves, and increases in certain functional groups such as smaller sized mixed feeders, frugivores, and nectarivores. Species diversity changes under increasing land use are variable (McKinney 2002; Fairbanks 2004; Shochat et al. 2006; Child et al. 2009; Van Rensburg et al. 2009; Coetzee et al. 2014), and our results support a general finding that functionally generalist taxa may increase in urban areas (Shochat et al. 2006; Van Rensburg et al. 2009; Evans et al. 2011). Primary productivity, and seasonality in primary productivity, contributes much to explaining patterns in species richness, abundance, and functional richness across all land uses.

When untransformed, as in the protected area, the Phalaborwa Sandy Mopaneveld vegetation type in our study region is mainly dominated by hardy and drought‐resident mopane trees (*Colophospermum mopane*), a gregariously growing species which may exclude other plants (Van Wyk and Van Wyk 2000). Mopane offers few nesting and foraging opportunities for resident birds (Hockey et al. 2005). Outside the protected area, human land‐use change generally increases the overall primary productivity and so promotes avian diversity there. The provisioning of water through irrigation and the promotion of gardening in the urban area will also increase primary productivity. Importantly, water provisioning in the urban area occurs throughout the year in a region with a pronounced wet and dry season (Fig. S5). Less seasonal variation in productivity because of water provisioning in an otherwise very seasonal landscape will contribute to increased richness and abundance and so promote species turnover (Hurlbert and Haskell 2003). The role of spatial heterogeneity as a driver of species richness across taxa, biomes, and spatial scales is well established (Stein et al. 2014), but the role of temporal heterogeneity is more controversial (Menge and Sutherland 1976; Rohde 1992; Archibald et al. 2010; Stein et al. 2014). Our study provides further evidence that a decline in temporal heterogeneity may increase species richness, abundance, and functional richness.

The quantification of functional diversity is generally undertaken for two reasons: first to identify how the patterns of functional trait composition vary between assemblages (e.g., Neuschulz et al. 2013), and second, to understand how such trait variation influences the functioning of ecosystems (Petchey and Gaston 2007). Here, the functional indices illustrated that the rural area contains the highest diversity of functional traits, despite the absence of traits unique to other land uses, such as large‐bodied ground nesters. The protected and matrix areas were characterized by high functional divergence, meaning that species with unique traits at high abundances in those land uses. The observed reduced abundance in large‐bodied ground nesters was likely conservative, as they are cryptic and difficult to detect using a point count methodology as they do not flush readily and are evasive. Line transect methods may have overcome this constraint, but were not used here owing to general obstructions across land uses. Similar declines of birds with large size outside of protected areas, particularly raptors, have been recorded elsewhere in African savannas (Herremans and Herremans‐Tonnoeyr 2000; Thiollay 2007).

However, the ecological implications of this functional change in our study region are less clear (Devictor et al. 2008; Clavel et al. 2011; O'Gorman et al. 2011). The functional roles of birds in ecosystems are increasingly recognized, but a comprehensive mechanistic understanding of the consequences of declines of specific groups in different ecosystems is still lacking (e.g., Sekercioglu 2006). Work on plants (Díaz et al. 2007), and food webs (O'Gorman et al. 2011), suggests that functional declines alter ecosystem service delivery, but the consequences of the functional loss for vertebrates nonetheless remains less thoroughly explored (Díaz et al. 2013). Body size is a fundamental species trait that affects a range of ecological properties such as abundance structure (Lewis et al. 2008) and life‐history strategies (Rohwer et al. 2009), as well as processes such as the structure and dynamics of food webs across multiple scales of organization. In consequence, functionally unique species from a body size perspective may be instrumental in linking biodiversity and ecosystem processes with landscape change (Loreau 2010; O'Gorman et al. 2011).

In conclusion, we found that different land uses in our study region have different biodiversity values in terms of bird assemblage composition. The findings support a growing consensus that the extent to which protected areas retain biodiversity varies with local scale factors (Evans et al. 2006; Gaston et al. 2008; Greve et al. 2011; Geldmann et al. 2013; Coetzee et al. 2014). Here we found that anthropogenic land‐use change has promoted the diversity of avian species, with one plausible influence being an increase in productivity and decrease in seasonality in productivity in the landscape. Habitats that are stressful to organisms may thus be altered by land‐use change to allow larger diversity for a suite of species, but may involve functional homogenization (Devictor et al. 2008; Clavel et al. 2011; O'Gorman et al. 2011), in terms of the loss of functionally unique species, although the ubiquity of this pattern remains to be examined. In consequence, in contrast to some views (e.g., Thomas 2013), our study shows that an increase in biodiversity by anthropogenic alteration cannot simply be interpreted as a positive outcome. Here, the increase in avian diversity came at the expense of species with unique traits (see also discussion in Mayfield et al. 2010). Elsewhere it has also been shown that even the increase of a single native species affected by anthropogenic habitat alteration has introduced substantial ecological dysfunction (Maron et al. 2013). Much still needs to be done to understand how general ecosystem effects of changes in species with particular traits are likely to be (Díaz et al. 2013), but it is clear that the simple interpretation that greater abundance of some groups of species in a landscape is somehow better is not necessarily correct.

## Conflict of Interest

The authors declare no conflict of interest.

## Supporting information


**Table S1.** GLM models for all land‐uses across seasons and sampling events.
**Table S2** Exemplar species from emergent species clusters.
**Table S3.** Indicator values (IndVals) for species from four land‐uses.
**Table S4.** Results from a general linear model comparing functional richness, eveness and diversity across all land‐uses as a function of primary productivity explanatory variables (mean NDVI and seasonal NDVI), latitude and longitude.
**Figure S1.** Species rarefaction curves.
**Figure S2.** Trait dendrogram for all species analysed.
**Figure S3.** Correlation between observed density and modelled density.
**Figure S4.** Best fit detection models for the seven surrogate species groups.
**Figure S5.** Averaged monthly rainfall data for Phalaborwa.
**Figure S6.** Raw species abundances.
**Figure S7.** Dendrograms for beta diversity.
**Figure S8.** Body size frequency distribution histograms.
**Appendix S1.** Mean abundance and species names for all bird species.

## References

[ece32389-bib-0001] Archibald, S. B. , W. H. Bossert , D. R. Greenwood , and B. D. Farrell . 2010 Seasonality, the latitudinal gradient of diversity, and Eocene insects. Paleobiology 36:374–398.

[ece32389-bib-0002] Baselga, A. 2012 The relationship between species replacement, dissimilarity derived from nestedness, and nestedness. Glob. Ecol. Biogeogr. 21:1223–1232.

[ece32389-bib-0003] Baselga, A. , and C. D. L. Orme . 2012 Betapart: an R package for the study of beta diversity. Methods Ecol. Evol. 3:808–812.

[ece32389-bib-0004] Biswas, S. R. , and A. U. Mallik . 2011 Species diversity and functional diversity relationship varies with disturbance intensity. Ecosphere 2:1–10.

[ece32389-bib-0005] Brown, A. M. , D. I. Warton , and N. R. Andrew . 2014 The fourth‐corner solution – using predictive models to understand how species traits interact with the environment. Methods Ecol. Evol. 5:344–352.

[ece32389-bib-0006] Cadotte, M. W. , K. Carscadden , and N. Mirotchnick . 2011 Beyond species: functional diversity and the maintenance of ecological processes and services. J. Appl. Ecol. 48:1079–1087.

[ece32389-bib-0007] Cardinale, B. J. , J. E. Duffy , A. Gonzalez , D. U. Hooper , C. Perrings , P. Venail , et al. 2012 Biodiversity loss and its impact on humanity. Nature 486:59–67. 22678280 10.1038/nature11148

[ece32389-bib-0008] Child, M. F. , G. S. Cumming , and T. Amano . 2009 Assessing the broad‐scale impact of agriculturally transformed and protected area landscapes on avian taxonomic and functional richness. Biol. Conserv. 142:2593–2601.

[ece32389-bib-0009] Chown, S. L. 2010 Temporal biodiversity change in transformed landscapes: a southern African perspective. Philos. Trans. R. Soc. B Biol. Sci. 365:3729–3742. 10.1098/rstb.2010.0274 PMC2982005 20980320

[ece32389-bib-0010] Clarke, K. R. , and R. M. Warwick . 2001 Primer‐E. Multivariate statistics for ecologists. Available at: http://www.primer-e.com (Accessed 30 January 2016).

[ece32389-bib-0011] Clavel, J. , R. Julliard , and V. Devictor . 2011 Worldwide decline of specialist species: toward a global functional homogenization? Front. Ecol. Environ. 9:222–228.

[ece32389-bib-0012] Clergeau, P. , J. L. Savard , G. Mennechez , and G. Falardeau . 1998 Bird abundance and diversity along and urban‐rural gradient: a comparative study between two cities on different continents. Condor 100:413–425.

[ece32389-bib-0013] Coetzee, B. W. T. , P. C. Le Roux , and S. L. Chown . 2013 Scale effects on the body size frequency distributions of African birds: patterns and potential mechanisms. Glob. Ecol. Biogeogr. 22:380–390.

[ece32389-bib-0014] Coetzee, B. W. T. , K. J. Gaston , and S. L. Chown . 2014 Local scale comparisons of biodiversity as a test for global protected area ecological performance: a meta‐analysis. PLoS One 9:e105824. 25162620 10.1371/journal.pone.0105824 PMC4146549

[ece32389-bib-0015] Colwell, R. K . 2005 EstimateS. Available at: http://viceroy.eeb.uconn.edu/estimates (Accessed 30 January 2016).

[ece32389-bib-0016] Devictor, V. , R. Julliard , D. Couvet , et al. 2007 The functional homogenization effect of urbanization on bird communities. Conserv. Biol. 21:741–751. 17531052 10.1111/j.1523-1739.2007.00671.x

[ece32389-bib-0017] Devictor, V. , R. Julliard , J. Clavel , et al. 2008 Functional biotic homogenization of bird communities in disturbed landscapes. Glob. Ecol. Biogeogr. 17:252–261.

[ece32389-bib-0018] Díaz, S. , S. Lavorel , F. de Bello , F. Quétier , K. Grigulis , and T. M. Robson . 2007 Incorporating plant functional diversity effects in ecosystem service assessments. Proc. Natl Acad. Sci. USA 104:20684–20689. 18093933 10.1073/pnas.0704716104 PMC2410063

[ece32389-bib-0019] Díaz, S. , A. Purvis , J. H. C. Cornelissen , G. M. Mace , M. J. Donoghue , R. M. Ewers , et al. 2013 Functional traits, the phylogeny of function, and ecosystem service vulnerability. Ecol. Evol. 3:2958–2975. 24101986 10.1002/ece3.601 PMC3790543

[ece32389-bib-0020] Dobrovolski, R. , R. D. Loyola , F. Guilhaumon , S. F. Gouveia , and J. A. F. Diniz‐Filho . 2013 Global agricultural expansion and carnivore conservation biogeography. Biol. Conserv. 165:162–170.

[ece32389-bib-0021] Dray, S. , and P. Legendre . 2008 Testing the species traits‐environment relationship: the fourth‐corner problem revisited. Ecology 89:3400–3412. 19137946 10.1890/08-0349.1

[ece32389-bib-0022] Dufrene, M. , and P. Legendre . 1997 Species assemblages and indicator species: the need for a flexible asymmetrical approach. Ecol. Monogr. 67:345–366.

[ece32389-bib-0023] Ellis, E. C. , and N. Ramankutty . 2008 Putting people in the map: anthropogenic biomes of the world. Front. Ecol. Environ. 6:439–447.

[ece32389-bib-0024] Ellis, E. C. , Klein Goldewijk, K. , Siebert, S. , Lightman, D. , and Ramankutty, N. 2010 Anthropogenic transformation of the biomes, 1700 to 2000. Glob. Ecol. Biogeogr. 19:589–606.

[ece32389-bib-0025] ESRI . 2014 Environmental systems resource institute. ESRI, Redlands, CA Available at: http://www.esri.com (Accessed 30 January 2016).

[ece32389-bib-0026] Evans, K. L. , J. J. D. Greenwood , and K. J. Gaston . 2005 Dissecting the species‐energy relationship. Proc. R. Soc. B Biol. Sci. 272:2155–2163. 10.1098/rspb.2005.3209 PMC1559954 16188604

[ece32389-bib-0027] Evans, K. L. , A. S. L. Rodrigues , S. L. Chown , and K. J. Gaston . 2006 Protected areas and regional avian species richness in South Africa. Biol. Lett. 2:184–188. 17148358 10.1098/rsbl.2005.0435 PMC1618914

[ece32389-bib-0028] Evans, K. L. , D. E. Chamberlain , B. J. Hatchwell , R. D. Gregory , and K. J. Gaston . 2011 What makes an urban bird? Glob. Change Biol. 17:32–44.

[ece32389-bib-0029] Fairbanks, D. H. K. 2004 Regional land‐use impacts affecting avian richness patterns in Southern Africa – insights from historical avian atlas data. Agric. Ecosyst. Environ. 101:269–288.

[ece32389-bib-0030] Foley, J. A. , R. Defries , G. P. Asner , C. Barford , G. Bonan , S. R. Carpenter , et al. 2007 Global consequences of land‐use. Science 309:570–574. 10.1126/science.1111772 16040698

[ece32389-bib-0031] Gaston, K. J. 2000 Global patterns in biodiversity. Nature 405:220–227. 10821282 10.1038/35012228

[ece32389-bib-0032] Gaston, K. J. , T. M. Blackburn , and K. K. Goldewijk . 2003 Habitat conversion and global avian biodiversity loss. Proc. R. Soc. B 270:1293–1300. 10.1098/rspb.2002.2303 PMC1691371 12816643

[ece32389-bib-0033] Gaston, K. J. , S. F. Jackson , L. Cantú‐Salazar , and G. Cruz‐Pinón . 2008 The ecological performance of protected areas. Annu. Rev. Ecol. Evol. Syst. 39:93–113.

[ece32389-bib-0034] Geldmann, J. , M. Barnes , L. Coad , M. Hockings , and N. D. Burgess . 2013 Effectiveness of terrestrial protected areas in reducing habitat loss and population declines. Biol. Conserv. 161:230–238.

[ece32389-bib-0035] Gerland, P. , A. E. Raftery , H. Sevčíková , N. Li , D. Gu , T. Spoorenberg , et al. 2014 World population stabilization unlikely this century. Science 346:234–237. 25301627 10.1126/science.1257469 PMC4230924

[ece32389-bib-0036] Greve, M. , S. L. Chown , B. J. van Rensburg , M. Dallimer , and K. J. Gaston . 2011 The ecological effectiveness of protected areas: a case study for South African birds. Anim. Conserv. 14:295–305.

[ece32389-bib-0037] Haberl, H. , K. H. Erb , F. Krausmann , V. Gaube , A. Bondeau , C. Plutzar , et al. 2007 Quantifying and mapping the human appropriation of net primary production in earth's terrestrial ecosystems. Proc. Natl Acad. Sci. 104:12942–12947. 17616580 10.1073/pnas.0704243104 PMC1911196

[ece32389-bib-0038] Herremans, M. , and D. Herremans‐Tonnoeyr . 2000 Land‐use and the conservation status of raptors in Botswana. Biol. Conserv. 94:31–41.

[ece32389-bib-0039] Hockey, P. A. R. , W. R. J. Dean , and P. G. Ryan . 2005 Roberts – Birds of southern Africa. The trustees of the John Voelcker Bird Book Fund, Cape Town.

[ece32389-bib-0040] Hurlbert, A. H. 2004 Species‐energy relationships and habitat complexity in bird communities. Ecol. Lett. 7:714–720.

[ece32389-bib-0041] Hurlbert, A. H. , and J. P. Haskell . 2003 The effect of energy and seasonality on avian species richness and community composition. Am. Nat. 161:83–97. 12650464 10.1086/345459

[ece32389-bib-0042] Kerr, J. T. , and M. Ostrovsky . 2003 From space to species: ecological applications for remote sensing. Trends Ecol. Evol. 18:299–305.

[ece32389-bib-0043] Laliberté, E. , and B. Shipley . 2013 Package “FD”. Available at: http://cran.r-project.org/web/packages/FD/FD.pdf (Accessed 30 January 2016).

[ece32389-bib-0044] Lewis, H. M. , R. Law , and A. J. McKane . 2008 Abundance–body size relationships: the roles of metabolism and population dynamics. J. Anim. Ecol. 77:1056–1062. 18547348 10.1111/j.1365-2656.2008.01405.x

[ece32389-bib-0045] de Lima, R. F. , M. Dallimer , P. W. Atkinson , and J. Barlow . 2013 Biodiversity and land‐use change: understanding the complex responses of an endemic‐rich bird assemblage. Divers. Distrib. 19:411–422.

[ece32389-bib-0046] Loreau, M. 2010 Linking biodiversity and ecosystems: towards a unifying ecological theory. Proc. Trans. R. Soc. B Biol. Sci. 365:49–60. 10.1098/rstb.2009.0155 PMC2842700 20008385

[ece32389-bib-0047] Luck, G. W. , S. Lavorel , S. McIntyre , and K. Lumb . 2012 Improving the application of vertebrate trait‐based frameworks to the study of ecosystem services. J. Anim. Ecol. 81:1065–1076. 22435774 10.1111/j.1365-2656.2012.01974.x

[ece32389-bib-0048] Luck, G. W. , A. Carter , and L. Smallbone . 2013 Changes in bird functional diversity across multiple land uses: interpretations of functional redundancy depend on functional group identity. PLoS One 8:e63671. 23696844 10.1371/journal.pone.0063671 PMC3656964

[ece32389-bib-0049] Maron, M. , M. J. Grey , C. P. Catterall , D. L. Oliver , M. F. Clarke , R. H. Loyn , et al. 2013 Avifaunal disarray due to a single despotic species. Divers. Distrib. 19:1468–1479.

[ece32389-bib-0050] Mayfield, M. M. , S. P. Bonser , J. W. Morgan , I. Aubin , S. McNamara , and P. Vesk . 2010 What does species richness tell us about functional trait diversity? Predictions and evidence for responses of species and functional trait diversity to land‐use change. Glob. Ecol. Biogeogr. 19:423–431.

[ece32389-bib-0051] McKinney, M. L. 2002 Urbanization, biodiversity, and conservation. Bioscience 52:883–890.

[ece32389-bib-0052] McKinney, M. L. 2006 Urbanization as a major cause of biotic homogenization. Biol. Conserv. 127:247–260.

[ece32389-bib-0053] Menge, B. A. , and J. P. Sutherland . 1976 Species diversity gradients: synthesis of the roles of predation, competition, and temporal heterogeneity. Am. Nat. 110:351–369.

[ece32389-bib-0054] Mori, A. S. , T. Furukawa , and T. Sasaki . 2013 Response diversity determines the resilience of ecosystems to environmental change. Biol. Rev. 88:349–364. 23217173 10.1111/brv.12004

[ece32389-bib-0055] Mouchet, M. , F. Guilhaumon , and N. Mason . 2008 Towards a consensus for calculating dendrogram‐based functional diversity indices. Oikos 117:794–800.

[ece32389-bib-0056] Mouillot, D. , N. A. J. Graham , S. Villeger , N. W. H. Mason , and D. R. Bellwood . 2013 A functional approach reveals community responses to disturbances. Trends Ecol. Evol. 28:167–177. 23141923 10.1016/j.tree.2012.10.004

[ece32389-bib-0057] Mucina, L. , and M. C. Rutherford . 2006 The vegetation of South Africa, Lesotho and Swaziland. South African National Biodiversity Institute, Pretoria.

[ece32389-bib-0058] Mulwa, R. K. , K. Böhning‐Gaese , and M. Schleuning . 2012 High bird species diversity in structurally heterogeneous farmland in western Kenya. Biotropica 44:801–809.

[ece32389-bib-0059] NASA . 2014 Land processes distributed active archive center. ASTER L1B. USGS/Earth resources observation and science (EROS) center, Sioux Falls, South Dakota Available at: https://lpdaac.usgs.gov (Accessed 30 January 2016).

[ece32389-bib-0060] Neuschulz, E. L. , M. Brown , and N. Farwig . 2013 Frequent bird movements across a highly fragmented landscape: the role of species traits and forest matrix. Anim. Conserv. 16:170–179.

[ece32389-bib-0061] Newbold, T. , J. P. W. Scharlemann , S. H. M. Butchart , Ç. H. Sekercioglu , R. Alkemade , H. Booth , et al. 2013 Ecological traits affect the response of tropical forest bird species to land‐use intensity. Proc. R. Soc. B 280:2012–2131. 10.1098/rspb.2012.2131 PMC3574433 23173205

[ece32389-bib-0062] Nieto, S. , P. Flombaum , and M. F. Garbulsky . 2015 Can temporal and spatial NDVI predict regional bird‐species richness? Glob. Ecol. Conserv. 3:729–735.

[ece32389-bib-0063] O'Gorman, E. J. , J. M. Yearsley , T. P. Crowe , M. C. Emmerson , U. Jacob , and O. L. Petchey . 2011 Loss of functionally unique species may gradually undermine ecosystems. Proc. R. Soc. B 278:1886–1893. 10.1098/rspb.2010.2036 PMC3097827 21106593

[ece32389-bib-0064] Pautasso, M. , K. Böhning‐Gaese , P. Clergeau , V. R. Cueto , M. Dinetti , E. Fernández‐Juricic , et al. 2011 Global macroecology of bird assemblages in urbanized and semi‐natural ecosystems. Glob. Ecol. Biogeogr. 20:426–436.

[ece32389-bib-0065] Peres, C. A. 2000 Effects of subsistence hunting on vertebrate community structure in Amazonian forests. Conserv. Biol. 14:240–253.

[ece32389-bib-0066] Petchey, O. L. , and K. J. Gaston . 2007 Dendrograms and measuring functional diversity. Oikos 116:1422–1426.

[ece32389-bib-0067] Pocock, M. J. O. 2011 Can traits predict species' vulnerability? A test with farmland passerines in two continents. Proc. R. Soc. B 278:1532–1538. 10.1098/rspb.2010.1971 PMC3081753 21047852

[ece32389-bib-0068] R Core Team . 2014 R: a language and environment for statistical computing. R Foundation for Statistical Computing, Vienna, Austria ISBN 3‐900051‐07‐0. Available at: http://www.R-project.org (Accessed 30 January 2016).

[ece32389-bib-0069] Rogers, A. M. , and S. Chown . 2014 Novel ecosystems support substantial avian assemblages: the case of invasive alien Acacia thickets. Divers. Distrib. 20:34–45.

[ece32389-bib-0070] Rohde, K. 1992 Latitudinal gradients in species diversity: the search for the primary cause. Oikos 65:514–527.

[ece32389-bib-0071] Rohwer, S. , R. E. Ricklefs , V. G. Rohwer , and M. M. Copple . 2009 Allometry of the duration of flight feather molt in birds. PLoS Biol. 7:e1000132. 19529759 10.1371/journal.pbio.1000132 PMC2690433

[ece32389-bib-0072] Schleuter, D. , M. Daufresne , F. Massol , and C. Argiller . 2010 A user's guide to functional diversity indices. Ecol. Monogr. 80:469–484.

[ece32389-bib-0073] Scholtz, C. H. , and S. L. Chown . 1993 Insect conservation and extensive agriculture: the savanna of southern Africa Pp. 75–95 *in* Gaston K., New T. R. and Samways M. J., eds. Perspectives on insect conservation. Intercept, Andover.

[ece32389-bib-0074] Schulze, C. H. , M. Waltert , P. J. Kessler , R. Pitopang , Shahabuddin, D. Veddeler , et al. 2004 Biodiversity indicator groups of tropical land‐use systems: comparing plants, birds, and insects. Ecol. Appl. 14:1321–1333.

[ece32389-bib-0075] Sekercioglu, C. H. 2006 Increasing awareness of avian ecological function. Trends Ecol. Evol. 21:464–471. 16762448 10.1016/j.tree.2006.05.007

[ece32389-bib-0076] Shochat, E. , P. S. Warren , S. H. Faeth , N. E. McIntyre , and D. Hope . 2006 From patterns to emerging processes in mechanistic urban ecology. Trends Ecol. Evol. 21:186–191. 16701084 10.1016/j.tree.2005.11.019

[ece32389-bib-0077] Sinclair, A. R. E. , S. A. R. Mduma , and P. Arcese . 2002 Protected areas as biodiversity benchmarks for human impact: agriculture and the Serengeti avifauna. Proc. R. Soc. B 269:2401–2405. 10.1098/rspb.2002.2116 PMC1691175 12495481

[ece32389-bib-0078] Stein, A. , K. Gerstner , and H. Kreft . 2014 Environmental heterogeneity as a universal driver of species richness across taxa, biomes and spatial scales. Ecol. Lett. 17:866–880. 24751205 10.1111/ele.12277

[ece32389-bib-0079] Storch, D. , K. L. Evans , and K. J. Gaston . 2005 The species‐area‐energy relationship. Ecol. Lett. 8:487–492. 21352452 10.1111/j.1461-0248.2005.00740.x

[ece32389-bib-0080] Sutherland, W. J. (1996) Ecological census techniques. Cambridge University Press, Cambridge.

[ece32389-bib-0081] Thiollay, J. 2007 Raptor declines in West Africa: comparisons between protected, buffer and cultivated areas. Oryx 41:322–329.

[ece32389-bib-0082] Thomas, C. D. 2013 The Anthropocene could raise biological diversity. Nature 502:7. 24091946 10.1038/502007a

[ece32389-bib-0083] Thomas, L. , S. T. Buckland , E. A. Rexstad , J. L. Laake , S. Strindberg , S. L. Hedley , et al. 2010 Distance software: design and analysis of distance sampling surveys for estimating population size. J. Appl. Ecol. 47:5–14. 20383262 10.1111/j.1365-2664.2009.01737.x PMC2847204

[ece32389-bib-0084] Tilman, D. 1999 Global environmental impacts of agricultural expansion: the need for sustainable and efficient practices. Proc. Natl Acad. Sci. USA 96:5995–6000. 10339530 10.1073/pnas.96.11.5995 PMC34218

[ece32389-bib-0085] du Toit, J. T. , B. H. Walker , and B. M. Campbell . 2003 The Kruger experience: ecology and management of savanna heterogeneity. Island Press, Washington, DC.

[ece32389-bib-0086] Trimble, M. J. , and R. J. van Aarde . 2012 Geographical and taxonomic biases in research on biodiversity in human‐modified landscapes. Ecoshpere 3:1–169.

[ece32389-bib-0087] Van Rensburg, B. J. , D. S. Peacock , and M. P. Robertson . 2009 Biotic homogenization and alien bird species along an urban gradient in South Africa. Landsc. Urban Plan 92:233–241.

[ece32389-bib-0088] Van Wyk, P. , and B. Van Wyk . 2000 Field guide to the trees of southern Africa. Random House Struik, Cape Town.

[ece32389-bib-0089] Villéger, S. , N. W. H. Mason , and D. Mouillot . 2008 New multi‐dimensional functional diversity indices for a multifaceted framework in functional ecology. Ecology 89:2290–2301. 18724739 10.1890/07-1206.1

[ece32389-bib-0090] Vitousek, P. M. , H. A. Mooney , J. Lubchenco , and J. M. Melillo . 1997 Human domination of earth's ecosystems. Science 277:494–499.

[ece32389-bib-0091] Wang, Y. , U. Naumann , S. T. Wright , and D. I. Warton . 2012 mvabund – an R package for model‐based analysis of multivariate abundance data. Methods Ecol. Evol. 3:471–474.

