# Supplementary material for: Land‐use change promotes avian diversity at the expense of species with unique traits
Source: Ecol Evol. 2016 Oct 5;6(21):7610–22. doi: 10.1002/ece3.2389 (PMC6093147; doi:10.1002/ece3.2389)
Supplement: Supplementary file 1 — Table S1. GLM models for all land‐uses across seasons and sampling events. Table S2 Exemplar species from emergent species clusters. Table S3. Indicator values (IndVals) for species from four land‐uses. Table S4. Results from a general linear model comparing functional richness, eveness and diversity across all land‐uses as a function of primary productivity explanatory variables (mean NDVI and seasonal NDVI), latitude and longitude. Figure S1. Species rarefaction curves. Figure S2. Trait dendrogram for all species analysed. Figure S3. Correlation between observed density and modelled density. Figure S4. Best fit detection models for the seven surrogate species groups. Figure S5. Averaged monthly rainfall data for Phalaborwa. Figure S6. Raw species abundances. Figure S7. Dendrograms for beta diversity. Figure S8. Body size frequency distribution histograms. Appendix S1. Mean abundance and species names for all bird species. [file ECE3-6-7610-s001.docx]

**Supplementary material**

**Land-use change promotes avian diversity at the expense of species with unique traits**

Bernard W. T. Coetzee* and Steven L. Chown

*Corresponding author: bwtcoetzee@gmail.com

**Table S1** GLM models for all land-uses across seasons and sampling events.

**Table S2** Exemplar species from emergent species clusters.

**Table S3** Indicator values (IndVals) for species from four land-uses.

**Fig. S1** Species rarefaction curves.

**Fig. S2** Trait dendrogram for all species analysed.

**Fig. S3** Correlation between observed density and modelled density.

**Fig. S4** Best fit detection models for the seven surrogate species groups.

**Fig. S5** Averaged monthly rainfall data for Phalaborwa.

**Fig. S6** Raw species abundances.

**Fig. S7** Dendrograms for beta diversity.

**Fig. S8** Body size frequency distribution histograms.

**Appendix S1** Mean abundance and species names for all bird species.

**Supplementary material**

**Table S1.** Results from generalized linear models comparing both species richness and abundances between land-uses as a function of sampling year, sampling month (a measure of seasonality) and land-uses.

|  |  |  |  |
| --- | --- | --- | --- |
|  | **Response variable:**  **Species richness** | |  |
|  | n = 280 | |  |
|  | Deviance explained | | 36.7% |
|  |  |  |  |
| Variables: | Slope | SE | P |
| **Intercept** | **1.85** | **0.791** | * |
| Land-use: protected area | -0.56 | 0.117 | *** |
| Land-use: rural | -0.23 | 0.103 | * |
| Land-use: urban | 0.65 | 0.097 | *** |
| Year | 0.03 | 0.074 |  |
| Month | -0.03 | 0.075 |  |
|  |  |  |  |
|  |  | |  |
|  | **Response variable:**  **Abundance** | |  |
|  | n = 280 | |  |
|  | Deviance explained | | 28.0% |
|  |  |  |  |
| Variables: | Slope | SE | P |
| **Intercept** | 3.77 | 1.535 | ***** |
| Land-use: protected area | -0.46 | 0.234 |  |
| Land-use: rural | -0.14 | 0.207 |  |
| Land-use: urban | 0.86 | 0.190 | ******* |
| Month | -0.24 | 0.146 |  |
| Year | -0.14 | 0.146 |  |
|  |  |  |  |
| **Significance codes:** |  |  |  |
| 0.001 = ***; 0.01 = **; 0.05 = * |  |  |  |
|  |  |  |  |
|  |  |  |  |

**Table S2.** Description of the functional groups (considering foraging, morphometric and nesting traits) from the trait dendrogram in Fig. S2 (Groups 1-7).

|  |  |  |  |  |
| --- | --- | --- | --- | --- |
| **Group** | **Typical characteristics** | **Exemplar species groups** |  |  |
|  |  |  |  |  |
| **1** | Large-bodied, Ground nesters | Francolins, Korhaans, Guineafowl |  |  |
|  |  |  |  |  |
| **2** | Small to medium bodied, mixed feeders, frugivores, hole nesters | Barbets, starlings, mousebirds |  |  |
|  |  |  |  |  |
| **3** | Small to medium bodied, granivores, mainly platform nesters | Buntings, Doves |  |  |
|  |  |  |  |  |
| **4** | Small to medium bodied, parasitic breeders or cup nesters, insectivores, nectarivores | Cuckoos, Sunbirds, Cisticolas |  |  |
|  |  |  |  |  |
| **5** | Small to medium bodied, mainly insectivores, mixed feeders, mixed breeders | Shrikes, Cuckoos, Flycatchers, Kingfishers |  |  |
|  |  |  |  |  |
| **6** | Small bodied, granivores, cup nesters | Weavers, waxbills, Firefinch |  |  |
|  |  |  |  |  |
| **7** | Medium bodied, insectivore/frugivores, mainly hole nesters | Hornbills |  |  |
|  |  |  |  |  |
|  |  |  |  |  |
| **Table S3.** Indicator values (IndVals) for species from four land-uses. IndVal describes the extent to which each species fulfils the criteria of specificity (uniqueness to a land-use), or fidelity (the frequency of occurrence within a land-use). It is expressed as a percentage, and higher values indicate that species is more representative of a particular land-uses. For illustrative purposes we selected species with IndVals higher than 60 % as indicator species for the land-uses.   \|  \|  \|  \|  \|  \|  \| \| --- \| --- \| --- \| --- \| --- \| --- \| \| **Protected** \| **Indval** \| **Matrix** \| **Indval** \| **Rural** \| **Indval** \| \| Sabota Lark \| 80 \| Blue Waxbill \| 100 \| Yellow fronted Canary \| 90 \| \| Laughing Dove \| 60 \| Laughing Dove \| 85 \| Blue Waxbill \| 85 \| \| Blue Waxbill \| 60 \| Red backed Shrike \| 75 \| Sabota Lark \| 80 \| \|  \|  \| Rattling Cisticola \| 70 \| Rattling Cisticola \| 70 \| \|  \|  \| Cape Turtle Dove \| 65 \| Tawny flanked Prinia \| 60 \| \|  \|  \| Yellow fronted Canary \| 60 \|  \|  \|  \| **Urban** \| **Indval** \| \| --- \| --- \| \| African Paradise Flycatcher \| 100 \| \| Kurrichane Thrush \| 100 \| \| Laughing Dove \| 100 \| \| Blue Waxbill \| 90 \| \| Dark Capped Bulbul \| 90 \| \| Forktailed Drongo \| 90 \| \| Red eyed Dove \| 90 \| \| African Hoopoe \| 80 \| \| Bronze Mannikin \| 80 \| \| Southern Masked Weaver \| 80 \| \| Yellow fronted Canary \| 80 \| \| Red Faced Mousebird \| 70 \| \| Southern Grey Headed Sparrow \| 60 \| \| Speckled Mousebird \| 60 \| \| White Bellied Sunbird \| 60 \|   **Table S4.** Results from a general linear model comparing functional richness, eveness and diversity across all land-uses as a function of primary productivity explanatory variables (mean NDVI and seasonal NDVI), latitude and longitude. Significant p values are denoted with an asterisk.   \| **Functional richness** \|  \|  \|  \| \| --- \| --- \| --- \| --- \| \|  \| Deviance explained \| 22.8% \|  \| \| Variables \| Coefficient \| Standard error \| P \| \| Intercept \| -0.24 \| 0.21 \| 0.25 \| \| Mean NDVI \| 0.01 \| 0.01 \| <0.001* \| \| Seasonal NDVI \| -0.01 \| 0.01 \| <0.001* \| \|  \|  \|  \|  \| \| **Functional eveness** \|  \|  \|  \| \|  \| Deviance explained \| 0.8% \|  \| \| Variables \| Coefficient \| Standard error \| P \| \| Intercept \| -0.25 \| 1.04 \| 0.80 \| \| Mean NDVI \| -0.01 \| 0.01 \| 0.93 \| \| Seasonal NDVI \| 0.01 \| 0.01 \| 0.94 \| \|  \|  \|  \|  \| \| **Functional diversity** \|  \|  \|  \| \|  \| Deviance explained \| 11.4% \|  \| \| Variables \| Coefficient \| Standard error \| P \| \| Intercept \| -0.32 \| 1.01 \| 0.74 \| \| Mean NDVI \| 0.01 \| 0.01 \| 0.85 \| \| Seasonal NDVI \| -0.01 \| 0.01 \| 0.77 \| \|  \|  \|  \|  \|   **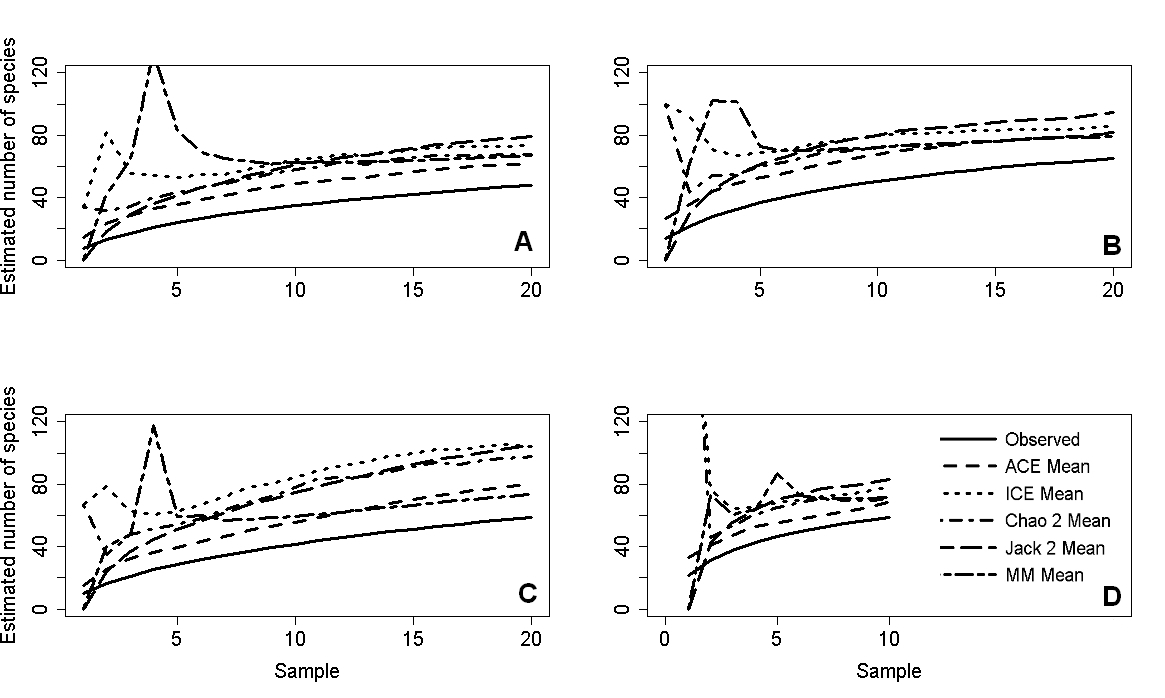**  **Fig. S1.** Species rarefaction curves by land-uses, for five major richness estimators and observed mean species richness (Observed). (A) protected (B) matrix (C) rural (D) urban areas. | | | |  |


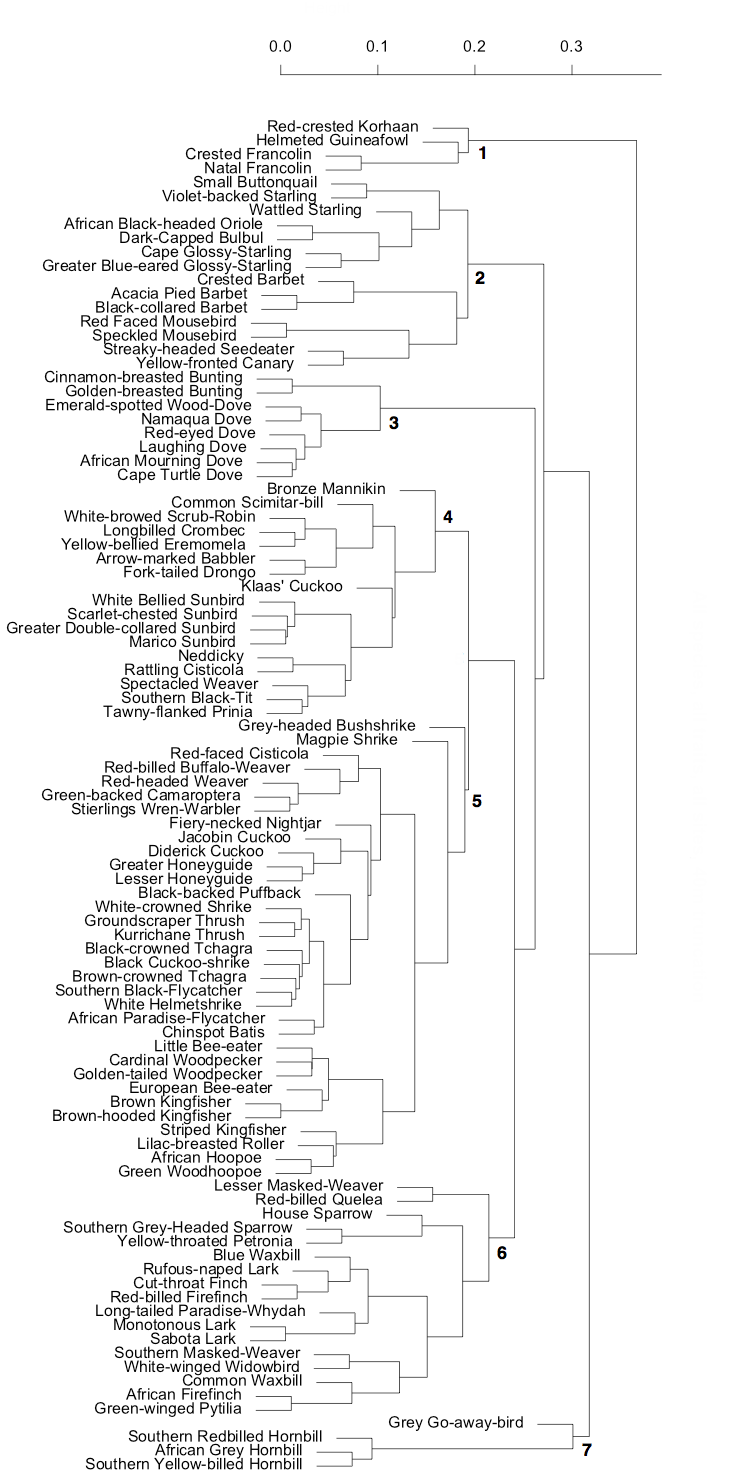


**Fig. S2**. Trait dendrogram for all species analysed. Major emergent groups are indicated for groups 1-7. Descriptions of these groups in terms of their typical foraging, morphometric and nesting behaviours are in Supplementary Table S2.

**
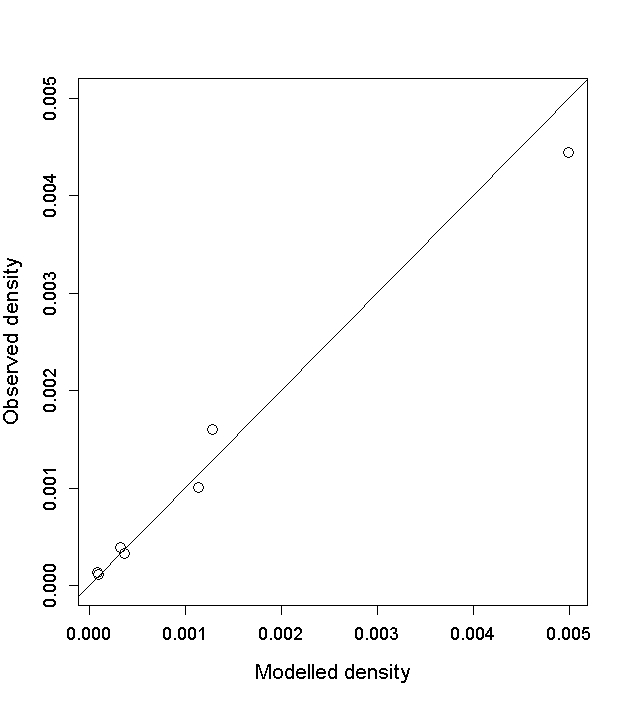
**

**Fig. S3.** Goodness of fit between observed density and modelled density is highly correlated (Pearson’s r = 0.994; p < 0.001), indicating that the influence of species detection is low, at least in this study region. The diagonal with a slope of one shows the hypothetical line of best fit if observed density = modelled density.


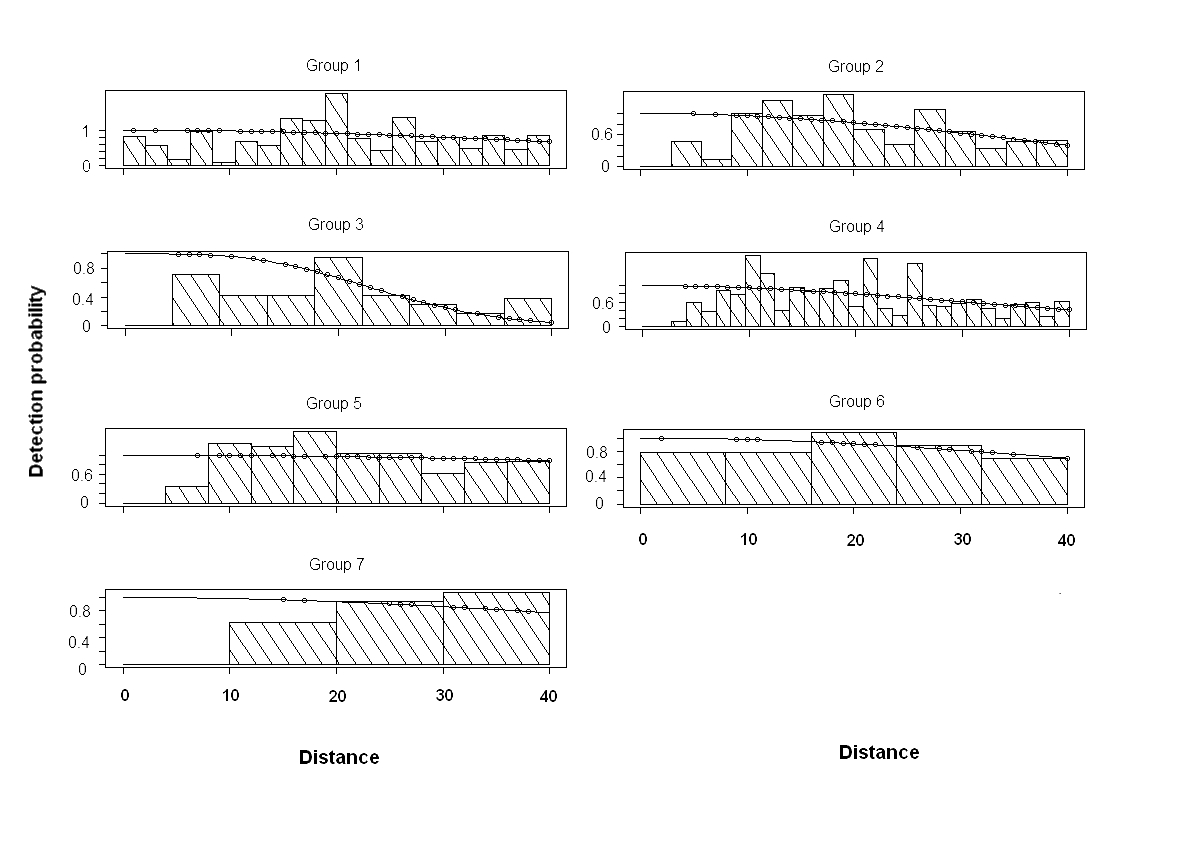


**Fig. S4.** Best fit detection models for the seven surrogate species groups examined, all showing monotonically decreasing detection functions. Best fit detection models for Groups 1-5 were uniform key with polynomial adjustments, while for Groups 6-7 were half-normal key with cosine adjustments, with the best fit models for all groups found at a 40 m truncation. Details of species groups are contained in Table S2.

Rainfall (mm)

**Fig. S5**. Averaged monthly rainfall data from 1967-2008 for Phalaborwa. Mean and standard deviations are shown. Source: South African Weather Service (www.weathersa.co.za) (Accessed January 2016).


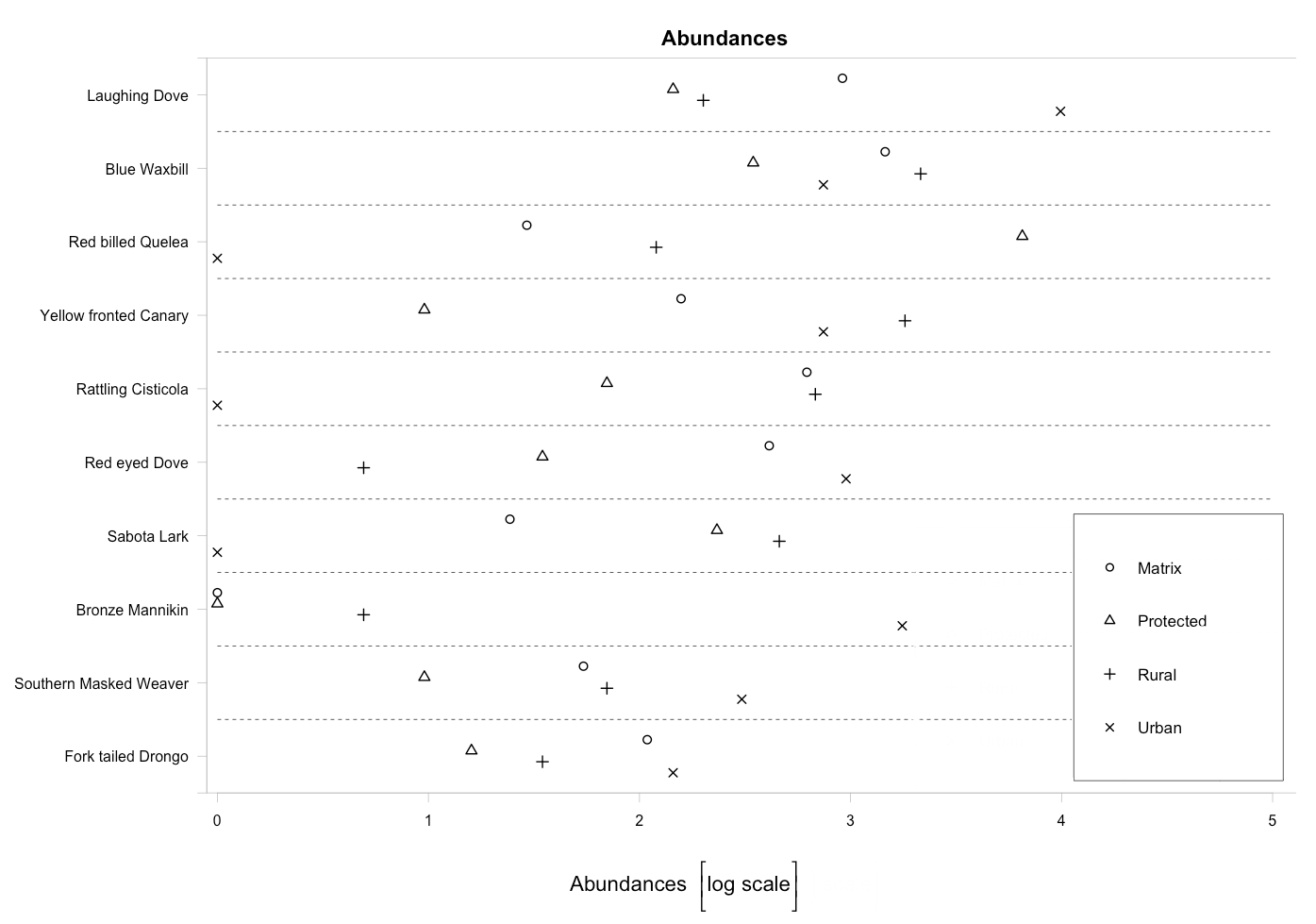


**Fig. S6.** Species abundances in each of the land-uses of the ten most prevalent species found across the total sampling area. Species names are in Appendix S1.


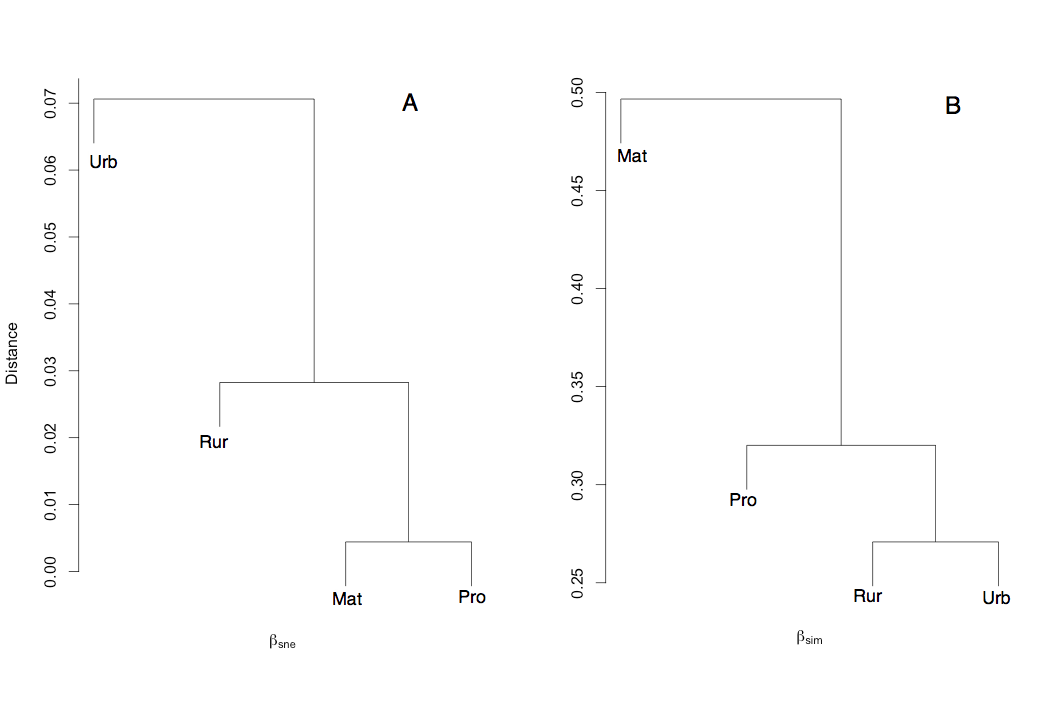


**Fig. S7.** Dendrograms for beta diversity decomposed into components of both the dissimilarity due to species replacement (A; ß_sne_) and dissimilarity due to nestedness within sites (B; ß_sim_). Pro = protected area; Mat = matrix area; Rur = rural area; Urb = urban area.


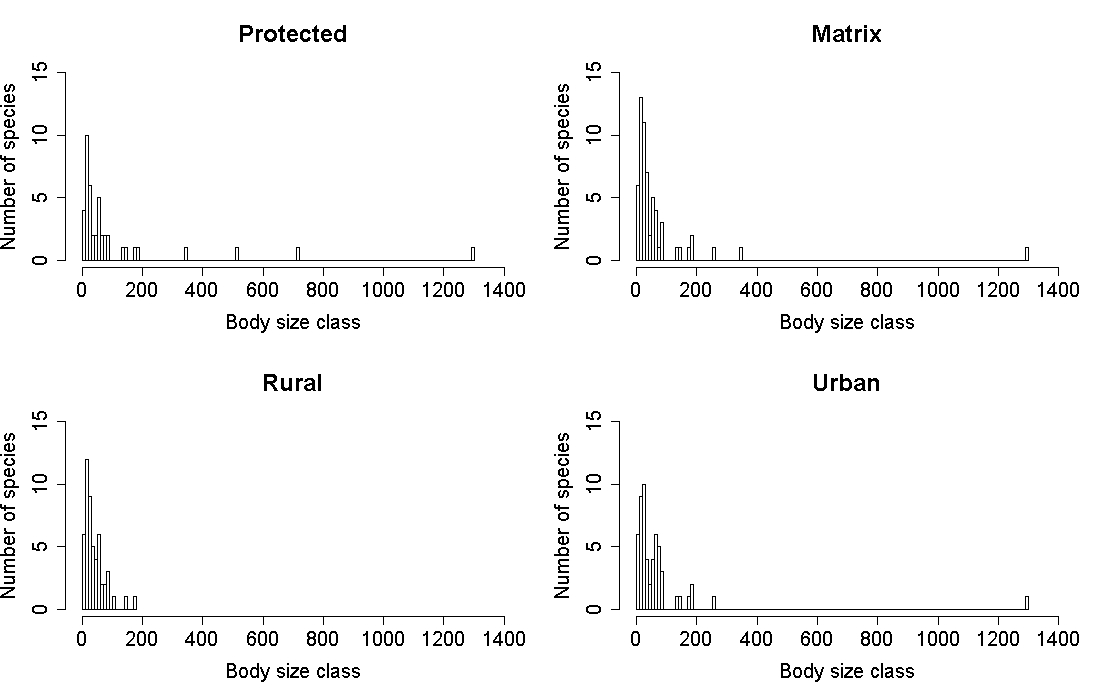


**Fig. S8.** Body size frequency distribution histograms for all species across land-uses. Body weight is in grams.

**Appendix S1.** Mean abundance for all bird species in all land-uses. Values in brackets indicate standard deviations. Feed. = foraging group (were F = Frugivore; M = Mixed feeder; I = Insectivore; G = Granivore; P = Predator; N = Nectarivore). M = Mean body mass in grams.

|  |  |  |  |  |  |  |  |  |
| --- | --- | --- | --- | --- | --- | --- | --- | --- |
| **ID** | **Common name** | **Scientific name** | **Protected** | **Matrix** | **Rural** | **Urban** | **Feed.** | **M** |
| 1 | Acacia Pied Barbet | *Tricholaema leucomelas* | 0 | 0.15(0.37) | 0.05(0.22) | 0 | F | 32.2 |
| 2 | African Black-headed Oriole | *Oriolus larvatus* | 0 | 0 | 0 | 0.1(0.32) | M | 66.1 |
| 3 | African Firefinch | *Lagonosticta rubricata* | 0 | 0.15(0.67) | 0 | 0 | M | 10.18 |
| 4 | African Grey Hornbill | *Tockus nasutus* | 0 | 0.2(0.52) | 0 | 0.2(0.42) | I/M | 182.3 |
| 5 | African Hoopoe | *Upupa africana* | 0 | 0.05(0.22) | 0.1(0.45) | 1.8(1.69) | I | 53.05 |
| 6 | African Mourning Dove | *Streptopelia decipiens* | 0 | 0 | 0 | 0.9(1.45) | G | 134 |
| 7 | African Paradise-Flycatcher | *Terpsiphone viridis* | 0 | 0.3(0.8) | 0.05(0.22) | 3.4(2.88) | I | 13.25 |
| 8 | Arrow-marked Babbler | *Turdoides jardineii* | 0.2(0.89) | 0 | 0 | 0.5(1.08) | I | 78.2 |
| 9 | Black Cuckoo-shrike | *Campephaga flava* | 0 | 0.05(0.22) | 0 | 0 | I | 32.3 |
| 10 | Black-backed Puffback | *Dryoscopus cubla* | 0 | 0.1(0.31) | 0 | 0.4(0.52) | I | 26.25 |
| 11 | Black-collared Barbet | *Lybius torquatus* | 0 | 0.15(0.49) | 0 | 0.1(0.32) | F | 51.7 |
| 12 | Black-crowned Tchagra | *Tchagra senegalus* | 0.05(0.22) | 0.05(0.22) | 0.1(0.45) | 0 | I | 53.5 |
| 13 | Blue Waxbill | *Uraeginthus angolensis* | 1.75(2) | 3.4(1.88) | 4.05(2.86) | 5(3.65) | G | 9.9 |
| 14 | Bronze Mannikin | *Spermestes cucullatus* | 0 | 0 | 0.15(0.67) | 7.4(7.69) | G | 9.9 |
| 15 | Brown-crowned Tchagra | *Tchagra australis* | 0.05(0.22) | 0.2(0.52) | 0.1(0.31) | 0 | I | 32.3 |
| 16 | Brown-hooded Kingfisher | *Halcyon albiventris* | 0 | 0 | 0.05(0.22) | 0.1(0.32) | P | 65.1 |
| 17 | Brubru | *Nilaus afer* | 0.2(0.62) | 0 | 0 | 0 | I | 19.4 |
| 18 | Cape Glossy-Starling | *Lamprotornis nitens* | 0 | 0 | 0.05(0.22) | 0.9(2.23) | M | 85.6 |
| 19 | Cape Turtle Dove | *Streptopelia capicola* | 0.55(0.94) | 1.8(1.99) | 0.25(0.55) | 0.6(0.7) | G | 142 |
| 20 | Cardinal Woodpecker | *Dendropicos fuscescens* | 0 | 0 | 0 | 0.1(0.32) | I | 26 |
| 21 | Chinspot Batis | *Batis molitor* | 0.3(0.57) | 0.15(0.37) | 0.15(0.49) | 0.6(1.26) | I | 11.65 |
| 22 | Cinnamon-breasted Bunting | *Emberiza tahapisi* | 0.15(0.37) | 0 | 0 | 0 | G | 13 |
| 23 | Common Scimitar-bill | *Rhinopomastus cyanomelas* | 0 | 0 | 0.05(0.22) | 0.1(0.32) | M | 28 |
| 24 | Common Waxbill | *Estrilda astrild* | 0 | 0.05(0.22) | 0 | 0 | M | 8.3 |
| 25 | Crested Barbet | *Trachyphonus vaillantii* | 0 | 0 | 0 | 0.3(0.67) | M | 69.45 |
| 26 | Crested Francolin | *Dendroperdix sephaena* | 0.15(0.49) | 0.6(1.14) | 0 | 0 | M | 341.5 |
| 27 | Cut-throat Finch | *Amadina fasciata* | 0 | 0 | 0.1(0.45) | 1.3(2.26) | M | 18 |
| 28 | Dark-Capped Bulbul | *Pycnonotus tricolor* | 0 | 0.3(0.92) | 0 | 4.5(3.47) | M | 37.3 |
| 29 | Diderick Cuckoo | *Chrysococcyx caprius* | 0 | 0.15(0.49) | 0.2(0.52) | 1(1.41) | I | 33 |
| 30 | Emerald-spotted Wood-Dove | *Turtur chalcospilos* | 0.1(0.45) | 0.15(0.37) | 0 | 0 | G | 60.6 |
| 31 | European Bee-eater | *Merops apiaster* | 0.15(0.37) | 0 | 0.05(0.22) | 0 | I | 56.6 |
| 32 | European Roller | *Coracias garrulus* | 0.1(0.31) | 0 | 0.05(0.22) | 0 | P | 146 |
| 33 | Fiery-necked Nightjar | *Caprimulgus pectoralis* | 0.05(0.22) | 0 | 0.1(0.31) | 0 | I | 52.5 |
| 34 | Fork-tailed Drongo | *Dicrurus adsimilis* | 0.35(0.67) | 1(1.38) | 0.55(0.76) | 2.3(1.77) | M | 40.3 |
| 35 | Golden-breasted Bunting | *Emberiza flaviventris* | 0.6(0.88) | 0.1(0.31) | 0.4(0.82) | 0.1(0.32) | G | 18.25 |
| 36 | Golden-tailed Woodpecker | *Campethera abingoni* | 0.05(0.22) | 0.15(0.37) | 0.05(0.22) | 0.1(0.32) | I | 68.5 |
| 37 | Greater Blue-eared Glossy-Starling | *Lamprotornis chalybaeus* | 0 | 0 | 0 | 0.6(1.9) | M | 86.5 |
| 38 | Greater Double-collared Sunbird | *Cinnyris afer* | 0 | 0 | 0.05(0.22) | 0 | N | 11.15 |
| 39 | Greater Honeyguide | *Indicator indicator* | 0.05(0.22) | 0 | 0 | 0 | I | 50.4 |
| 40 | Green Woodhoopoe | *Phoeniculus purpureus* | 0 | 0 | 0 | 0.7(1.64) | I | 74.25 |
| 41 | Green-backed Camaroptera | *Camaroptera brachyura* | 0 | 0.05(0.22) | 0 | 0 | I | 9.825 |
| 42 | Green-winged Pytilia | *Pytilia melba* | 0.1(0.45) | 0.05(0.22) | 0 | 0 | M | 15.4 |
| 43 | Grey Go-away-bird | *Corythaixoides concolor* | 0 | 0.35(0.75) | 0 | 0.8(1.62) | F | 258 |
| 44 | Grey-headed Bushshrike | *Malaconotus blanchoti* | 0 | 0 | 0 | 0.1(0.32) | P | 78.7 |
| 45 | Groundscraper Thrush | *Psophocichla litsipsirupa* | 0 | 0 | 0.2(0.89) | 0.2(0.42) | I | 74 |
| 46 | Helmeted Guineafowl | *Numida meleagris* | 0.4(1.79) | 0.1(0.31) | 0 | 0.2(0.63) | M | 1299 |
| 47 | House Sparrow | *Passer domesticus* | 0 | 0 | 0 | 2.6(4.43) | M | 25.8 |
| 48 | Icterine Warbler | *Hippolais icterina* | 0 | 0.05(0.22) | 0 | 0 | I | 13.2 |
| 49 | Jacobin Cuckoo | *Clamator jacobinus* | 0 | 0.05(0.22) | 0.05(0.22) | 0 | I | 81.5 |
| 50 | Klaas' Cuckoo | *Chrysococcyx klaas* | 0.05(0.22) | 0.1(0.31) | 0.1(0.31) | 0 | I | 27.4 |
| 51 | Kurrichane Thrush | *Turdus libonyanus* | 0 | 0.05(0.22) | 0 | 3.8(2.04) | I | 60.6 |
| 52 | Laughing Dove | *Streptopelia senegalensis* | 1.15(1.46) | 2.75(2.27) | 1.35(1.6) | 16(5.27) | G | 83.89 |
| 53 | Lesser Grey Shrike | *Lanius minor* | 0 | 0 | 0.05(0.22) | 0 | I | 46.5 |
| 54 | Lesser Honeyguide | *Indicator minor* | 0.05(0.22) | 0.05(0.22) | 0 | 0 | I | 28.2 |
| 55 | Lesser Masked-Weaver | *Ploceus intermedius* | 0 | 1.4(2.96) | 0 | 0.6(0.97) | M | 20.6 |
| 56 | Lesser Striped-Swallow | *Hirundo abyssinica* | 0 | 0 | 0 | 0.1(0.32) | I | 13.5 |
| 57 | Lilac-breasted Roller | *Coracias caudatus* | 0 | 0 | 0.25(0.64) | 0 | P | 110 |
| 58 | Little Bee-eater | *Merops pusillus* | 0.05(0.22) | 0.2(0.7) | 0.1(0.45) | 0 | I | 15.1 |
| 59 | Longbilled Crombec | *Sylvietta rufescens* | 0.05(0.22) | 0.45(0.76) | 0.05(0.22) | 0 | M | 11.6 |
| 60 | Long-tailed Paradise-Whydah | *Vidua paradisaea* | 0 | 0.1(0.31) | 0.1(0.45) | 0 | M | 20.4 |
| 61 | Magpie Shrike | *Corvinella melanoleuca* | 0.15(0.49) | 0.1(0.31) | 0 | 0 | P | 82.2 |
| 62 | Marico Sunbird | *Cinnyris mariquensis* | 0 | 0 | 0 | 0.4(0.7) | N | 11.5 |
| 63 | Monotonous Lark | *Mirafra passerina* | 0.05(0.22) | 0 | 0.05(0.22) | 0 | M | 24.7 |
| 64 | Namaqua Dove | *Oena capensis* | 0 | 0 | 0.05(0.22) | 0 | G | 40.59 |
| 65 | Natal Francolin | *Pternistis natalensis* | 0.15(0.67) | 0 | 0 | 0 | M | 516 |
| 66 | Neddicky | *Cisticola fulvicapilla* | 0.1(0.31) | 0.05(0.22) | 0.2(0.52) | 0.1(0.32) | I | 8.25 |
| 67 | Pale Flycatcher | *Bradornis pallidus* | 0 | 0 | 0.05(0.22) | 0 | I | 23.6 |
| 68 | Rattling Cisticola | *Cisticola chiniana* | 0.8(1.44) | 2.3(2.62) | 2.4(2.66) | 0 | I | 16.25 |
| 69 | Red Faced Mousebird | *Urocolius indicus* | 0 | 1.3(3.03) | 0.05(0.22) | 2.8(4.34) | F | 56.4 |
| 70 | Red-backed Shrike | *Lanius collurio* | 0.35(0.67) | 1(0.79) | 0.65(1.84) | 0 | I | 28.45 |
| 71 | Red-billed Buffalo-Weaver | *Bubalornis niger* | 0.05(0.22) | 0.65(2.25) | 0.1(0.45) | 0 | M | 76.4 |
| 72 | Red-billed Firefinch | *Lagonosticta senegala* | 0 | 0 | 0 | 0.1(0.32) | M | 9.27 |
| 73 | Red-billed Quelea | *Quelea quelea* | 6.65(24.51) | 0.5(2.24) | 1.05(3.55) | 0 | G | 18.6 |
| 74 | Red-crested Korhaan | *Lophotis ruficrista* | 0.1(0.31) | 0 | 0 | 0 | M | 714 |
| 75 | Red-eyed Dove | *Streptopelia semitorquata* | 0.55(0.76) | 1.9(5.28) | 0.15(0.49) | 5.6(6.19) | G | 176 |
| 76 | Red-faced Cisticola | *Cisticola erythrops* | 0 | 0 | 0.05(0.22) | 0 | I | 14.8 |
| 77 | Red-headed Weaver | *Anaplectes melanotis* | 0 | 0.2(0.52) | 0.1(0.45) | 0.4(0.7) | I | 22.6 |
| 78 | Rufous-naped Lark | *Mirafra africana* | 0 | 0 | 0.05(0.22) | 0 | M | 45.22 |
| 79 | Sabota Lark | *Calendulauda sabota* | 1.45(1.1) | 0.45(0.69) | 2(1.41) | 0 | M | 24.7 |
| 80 | Scarlet-chested Sunbird | *Chalcomitra senegalensis* | 0 | 0 | 0 | 0.4(0.97) | N | 12.43 |
| 81 | Small Buttonquail | *Turnix sylvaticus* | 0.1(0.31) | 0 | 0 | 0 | M | 54.25 |
| 82 | Southern Black-Flycatcher | *Melaenornis pammelaina* | 0 | 0 | 0 | 0.3(0.67) | I | 30.2 |
| 83 | Southern Black-Tit | *Parus niger* | 0.4(0.88) | 0.8(1.15) | 0.45(0.83) | 0.2(0.63) | M | 21.5 |
| 84 | Southern Grey-Headed Sparrow | *Passer diffusus* | 0.2(0.52) | 0.2(0.52) | 0.3(0.73) | 1.2(1.55) | M | 24.2 |
| 85 | Southern Masked-Weaver | *Ploceus velatus* | 0.25(1.12) | 0.7(1.56) | 0.8(1.77) | 3.3(2.41) | M | 31.05 |
| 86 | Southern Redbilled Hornbill | *Tockus erythrorhynchus* | 0.05(0.22) | 0.7(0.98) | 0 | 0 | I/M | 139 |
| 87 | Southern Yellow-billed Hornbill | *Tockus leucomelas* | 0.05(0.22) | 0.35(0.93) | 0 | 0.2(0.63) | I/M | 189.5 |
| 88 | Speckled Mousebird | *Colius striatus* | 0 | 0.45(1.23) | 0.1(0.31) | 2.1(2.85) | F | 51.45 |
| 89 | Spectacled Weaver | *Ploceus ocularis* | 0 | 0.05(0.22) | 0 | 0.4(0.84) | M | 25.2 |
| 90 | Spotted Flycatcher | *Muscicapa striata* | 0.1(0.31) | 0.05(0.22) | 0.4(0.75) | 0.4(0.97) | I | 15.9 |
| 91 | Stierlings Wren-Warbler | *Calamonastes stierlingi* | 0 | 0.1(0.31) | 0 | 0 | I | 13.15 |
| 92 | Streaky-headed Seedeater | *Crithagra gularis* | 0 | 0.05(0.22) | 0 | 0.1(0.32) | M | 16 |
| 93 | Striped Kingfisher | *Halcyon chelicuti* | 0 | 0.05(0.22) | 0 | 0 | P | 44.5 |
| 94 | Tawny-flanked Prinia | *Prinia subflava* | 0.05(0.22) | 0.45(0.6) | 1.25(1.68) | 1.2(1.81) | M | 8.649 |
| 95 | Violet-backed Starling | *Cinnyricinclus leucogaster* | 0.05(0.22) | 0 | 0.6(1.1) | 0.5(1.27) | M | 44 |
| 96 | Wattled Starling | *Creatophora cinerea* | 0 | 0 | 0 | 0.6(1.9) | M | 73.05 |
| 97 | White Bellied Sunbird | *Cinnyris talatala* | 0 | 0.4(0.6) | 0.8(1.24) | 1.3(1.42) | N | 8.1 |
| 98 | White Helmetshrike | *Prionops plumatus* | 0 | 1.3(3.31) | 0.1(0.45) | 0 | I | 33.8 |
| 99 | White-browed Scrub-Robin | *Cercotrichas leucophrys* | 0.45(0.83) | 0.45(0.51) | 0.6(0.94) | 0.1(0.32) | M | 16.5 |
| 100 | White-crowned Shrike | *Eurocephalus anguitimens* | 0 | 0.15(0.67) | 0 | 0 | I | 69.05 |
| 101 | White-winged Widowbird | *Euplectes albonotatus* | 0 | 0.1(0.45) | 0.35(0.93) | 0 | M | 21.15 |
| 102 | Willow Warbler | *Phylloscopus trochilus* | 0.15(0.37) | 0.05(0.22) | 0.2(0.7) | 0 | I | 8.7 |
| 103 | Woodland Kingfisher | *Halcyon senegalensis* | 0 | 0 | 0 | 0.8(1.03) | P | 74.5 |
| 104 | Yellow-bellied Eremomela | *Eremomela icteropygialis* | 0.15(0.49) | 0 | 0.15(0.49) | 0 | M | 7.55 |
| 105 | Yellow-fronted Canary | *Crithagra mozambicus* | 0.25(0.55) | 1.2(1.51) | 3.75(2.59) | 5(4.37) | G | 11.95 |
| 106 | Yellow-throated Petronia | *Petronia superciliaris* | 0 | 0 | 0 | 0.2(0.63) | M | 24 |
|  |  |  |  |  |  |  |  |  |
